# Cannabis Hunger Games: nutrient stress induction in flowering stage – impact of organic and mineral fertilizer levels on biomass, cannabidiol (CBD) yield and nutrient use efficiency

**DOI:** 10.3389/fpls.2023.1233232

**Published:** 2023-09-19

**Authors:** Danilo Crispim Massuela, Sebastian Munz, Jens Hartung, Peteh Mehdi Nkebiwe, Simone Graeff-Hönninger

**Affiliations:** ^1^Agronomy, Institute of Crop Science, University of Hohenheim, Stuttgart, Germany; ^2^Biostatistics, Institute of Crop Science, University of Hohenheim, Stuttgart, Germany; ^3^Department of Fertilization and Soil Matter Dynamics, Institute of Crop Science, University of Hohenheim, Stuttgart, Germany

**Keywords:** cannabidiol, nutrient stress, indoor cultivation, organic fertilization, mineral fertilization, environmental impact, nutrient use efficiency, medicinal cannabis

## Abstract

Indoor medicinal cannabis cultivation systems enable year-round cultivation and better control of growing factors, however, such systems are energy and resource intensive. Nutrient deprivation during flowering can trigger nutrient translocation and modulate the production of cannabinoids, which might increase agronomic nutrient use efficiency, and thus, a more sustainable use of fertilizers. This experiment compares two fertilizer types (mineral and organic) applied in three dilutions (80, 160 and 240 mg N L^−1^) to evaluate the effect of nutrient deprivation during flowering on biomass, Cannabidiol (CBD) yield and nutrient use efficiency of N, P and K. This is the first study showing the potential to reduce fertilizer input while maintaining CBD yield of medicinal cannabis. Under nutrient stress, inflorescence yield was significantly lower at the final harvest, however, this was compensated by a higher CBD concentration, resulting in 95% of CBD yield using one-third less fertilizer. The higher nutrient use efficiency of N, P, and K in nutrient-deprived plants was achieved by a larger mobilization and translocation of nutrients increasing the utilization efficiency of acquired nutrients. The agronomic nutrient use efficiency of CBD yield – for N and K – increased 34% for the organic fertilizers and 72% for the mineral fertilizers comparing the dilution with one-third less nutrients (160) with the highest nutrient concentration (240). Differences in CBD yield between fertilizer types occurred only at the final harvest indicating limitations in nutrient uptake due to nutrient forms in the organic fertilizer. Our results showed a lower acquisition and utilization efficiency for the organic fertilizer, proposing the necessity to improve either the timing of bio-availability of organic fertilizers or the use of soil amendments.

## 1 Introduction

Medicinal cannabis (*Cannabis sativa* L.) is a flourishing crop with increasing interest in the horticultural and medical fields. With the shift in regulation in several countries, diverse growing operations are taking place worldwide (Decorte and Potter, 2015). Indoor medicinal cannabis cultivation systems enable year-round cultivation, especially in temperate regions. Such systems offer more control of the overall production, with a higher degree of specialization, modification of the environmental conditions (i.e., light, air circulation, humidity and temperature) and plant abiotic stress induction (Cervantes, 2006; Jin et al., 2019; Malík et al., 2021). However, indoor systems are energy- and resource-intensive (Madhusoodanan, 2019; Wartenberg et al., 2021) while negatively affecting the environment through water, air and land pollution due to high water and fertilizer consumption (Pagnani et al., 2018; Zheng et al., 2021).

Indoor medicinal cannabis is commonly grown either in pots with substrate (generally peat or coir based) or soilless (e.g., rockwool, hydroponics) and rarely cultivated on raised beds or living soil (Malík et al., 2021; Nemati et al., 2021). The majority of nutrients are provided via fertigation systems and drip irrigation, enabling systems with higher control over the amount of water and nutrients provided to the plants. Liquid nutrients are either organic or mineral. Mineral fertilizers are chemical salts that are soluble and readily available to plants, thus increasing nutrient uptake, utilization and use efficiencies, but can cause salt accumulation in the root zone when provided in excess (Maschner, 1986). Excess of nutrients in the soil can decrease cannabinoid yield by reducing inflorescence biomass and cannabinoids concentration (Caplan et al., 2017a; Saloner and Bernstein, 2021). Mineral nutrients and other agrochemicals are often applied indiscriminately in agriculture, generating undoubtedly environmental consequences, entering water bodies by surface runoff and leaching from agricultural lands (Brownlie et al., 2021) and as a waste product from single-use substrates in indoor facilities, that end up in landfills, often mixed with disposable plastic containers used as pots, which is alarmingly preferred by the majority of commercial indoor cultivation facilities.

Due to market shifts, mineral fertilizer prices have dramatically increased over the past two years (Eisa et al., 2022). Prices for macronutrients such as nitrogen and phosphorous compounds put pressure to secure inputs for the cannabis cultivation sector (Drotleff, 2022). Currently, cannabis farmers in the U.S. seek alternative organic solutions, such as animal manure and compost teas (Drotleff, 2022). Such fertilizers are often locally available and do not depend on international supply chains and industrialization like mineral fertilizers. Nonetheless, the conversion from mineral to organic is generally challenging, as organic fertilization requires adaptation of cultivation systems, including the addition of soil amendments and correct timing of fertilizer applications for nutrients to be available to plants.

Organic fertilizers can be produced from a diversity of biological components and are assumed to be a more sustainable type of fertilizer, as they can be locally produced and use less energy compared to mineral fertilizers (Chang et al., 2007). However, the exact amount of nutrients provided to the plant is difficult to quantify as biological interactions in the rhizosphere are complex (Lowenfels, 2013). The availability of nutrients to the plant depends on the conversion by bacteria, fungi and other microorganisms (Lowenfels and Lewis, 2010; Jacoby et al., 2017), which can also present challenges with soil pathogens in comparison to soilless and mineral fertilizer systems (Nemati et al., 2021).

Multiple studies indicate that the metabolism of phytocannabinoids is highly sensitive to mineral (Bernstein et al., 2019; Malík et al., 2021) and organic nutrition (Caplan et al., 2017a; Caplan et al., 2017b; Bruce et al., 2022). More research has been conducted with liquid mineral fertilizer, suggesting positive effects on inflorescence biomass to N and P while conclusions for K are still limited and controversial (Bevan et al., 2021). The response of plant growth to fertilizer amount often follows a convex to bell-shaped curve, in which plant growth responds positively until an optimum fertilizer amount and then decreases at higher rates. Values for nutrient recommendations reported in literature for medicinal cannabis are highly variable on the cultivation system. Based on a surface response model, inflorescence yield in a hydroponic system with continuous mineral fertilizer responded best in the ranges of 160–230 mg N L^−1^ (estimated optimum at 194 mg N L^−1^) and 40–80 mg P L^−1^ (estimated optimum at 59 mg P L^−1^), while no response to K within the range of 60–340 mg K L^−1^ was observed (Bevan et al., 2021). Even less information is available for liquid organic fertilizers. In the experiments from Caplan et al., (2017a; 2017b), the recommended fertilizer concentration for organic fertilizer was 389 mg N L^−1^ (4.0N-1.3P-1.7K) for the vegetative growth stage and a rate of 212–261 mg N L^−1^ (2.0N-0.8P-3.3K) for the flowering stage. In flowering, there was a positive correlation between fertilizer rate and inflorescence yield but a negative correlation to tetrahydrocannabinolic acid (THCA) concentration, indicating a dilution effect of THCA with increasing yield.

To avoid nutrient deficiency symptoms, the correct supply of nutrients at the relevant stages of development is important, as under- and overfertilization of macronutrients result in diminished growth and inflorescence quality (Bernstein et al., 2019; Landi et al., 2019; Bevan et al., 2021; Ii et al., 2021). Nevertheless, it is known that abiotic stress like nutrient deprivation can trigger the production of secondary metabolites in plants, vital for plant defense and survival (Aguirre-Becerra et al., 2021). Results from Saloner and Bernstein (2021) report increase of terpenoids and cannabinoids under N deprivation (< 160 mg L^−1^). Authors suggest that secondary metabolites may also play an essential role in non-defensive plant function, in an attempt to improve competitive plant abilities in the struggle for vital resources such as water and nutrients (Wink, 2008). Yet, results are not conclusive if stress can increase cannabinoid yield, without compromising biomass yield, being instead a simple dilution effect. Consequently, it is paramount to research if the controlled induction of nutrient stress during flowering can increase the production of cannabinoids, leading to a higher nutrient agronomic use efficiency without reducing biomass, thus optimizing the efficiency of cannabinoids’ yield per plant/area for the amount of nutrients supplied.

Nutrient use efficiency (NUE) can be separated into uptake efficiency (NUpE) and utilization efficiency (NUtE) relating nutrient uptake to plant biomass or biomass of harvested organ to nutrient content of the plant, respectively (Congreves et al., 2021). A higher NUE can be achieved either by increasing nutrient uptake or higher re-mobilization and translocation into harvested organs (Hirose, 2011). NUpE correlates with nutrient sources and assimilation in roots, thus varying based on fertilizer type and form (Menz et al., 2018). NUtE can increase when uptake is limited, by the utilization of nutrients already acquired in plant organs (Ye et al., 2022). Additionally, under nutrient deprivation, plant elicitors and stressors can produce metabolic responses (eustress) that alter signaling pathways, and can favor the synthesis of terpenoids and phenolic compounds in plants (Aguirre-Becerra et al., 2021). Nutrient agronomic use efficiency (AE) is the contribution of fertilizer towards yield, compared to a non-fertilized control (Congreves et al., 2021). For medicinal cannabis production, AE for inflorescences dry matter (AE_inflorescences_) and the yield of Cannabidiol (CBD) (AE_CBDyield_) are the most relevant factors to guide nutrient use efficiency evaluation. The effect of organic or mineral fertilizer types and their concentrations on NUpE, NUtE and AE has not been reported yet for medicinal cannabis.

The Convention on Biological Diversity proposed to reduce excess nutrient pollution by 50% by 2030 (CBD, 2022), so the optimized use of mineral fertilizers in current cannabis commercial production is essential to reduce the environmental impact, with the reduction of mineral fertilizer input or the replacement by organic fertilizers. Given the limited number of studies for medicinal cannabis fertilization strategies and the relative importance of N, P and K on plant growth, the comparison of mineral and organic fertilizers and the effect of nutrient deprivation on cannabinoid production is crucial to establishing more accurate recommendations of fertilizer regimes.

The aim of this study is to investigate the effect of nutrient deprivation during flowering stage for mineral and organic fertilizer dilutions in order to evaluate whether fertilizer inputs can be reduced without penalizing CBD yield.

To reach this aim, the objectives of this study are (i) to quantify the dynamics in plant growth, inflorescence yield, CBD concentration and resulting CBD yield during the flowering stage, (ii) to investigate nutrient (N, P, K) re-mobilization and translocation among plant organs; and, (iii) to calculate resulting nutrient use efficiencies.

## 2 Materials and methods

An indoor experiment was performed at the University of Hohenheim (Stuttgart, Germany) between October 2021 and January 2022. Plants from a CBD-rich cannabis chemotype III genotype provided by AiFame (Wald-Schönengrund, Switzerland) were grown in a cultivation room inside the greenhouse complex, which is built with double insulated glass and automated environmental regulation systems. Air temperature and humidity were constantly monitored. During the cultivation period, the daily mean air temperatures varied from 17.9–29.4 °C and relative humidity was between 26.0 and 75.7%. Daily values of average, minimum and maximum air temperature and relative humidity are presented in (**Supplementary Figure 1**).

### 2.1 Cultivation methods

Plants were generated by cloning standardized stock mother plants. Clones were derived from cuttings of the apical tip of upper branches, then dipped into Rhizopon AA 1% powder (Rhizopon, Rijndijk, Netherlands) and transferred into EazyPlugs (3.5 cm × 3.5 cm × 3.5 cm) (Goirle, Netherlands). These cuttings were cultivated in a nursery greenhouse, which was humidified to above 80% and ventilated for proper air circulation. After 14 days, rooted cuttings were transplanted into round pots of 14 cm diameter containing 255 ± 5 g of Klasmann Substrate-5 + Green Fiber (Klasmann-Deilmann GmbH, Geeste, Germany), mixed with 15% perlite of PerligranR Extra (KNAUF, Germany). The day of transplanting was considered as the beginning of the experiment, being the initial day after planting (0 DAP). An Arbuscular Mycorrhiza Fungi (AMF) soluble mixture “Mykorrhiza Soluble” (Tyroler Glueckspilze, Austria) solution of 1.5 g L^−1^ was provided to the substrate during the first week, in total 350 ml per plant. After two weeks (15 DAP), each plant was transferred to a 4 L square pots containing 1070 ± 5 g of the same substrate composition as described above. Pots were placed in four rows each with nine pots on horticultural tables (1.0 m × 2.5 m) resulting in a density of 14.4 plants m^−^².

During the vegetative period, plants were pruned via an apical cut (topping) by the seventh internode, resulting in plants with six side shoots. By the vegetative period, the length of the photoperiod was 18 h, provided by ceramic metal halide lamps (CHD Agro 400W; DH Licht GmbH, Wülfrath, Germany). The total, vegetative duration after planting was 36 days. During the generative period, photoperiod was 12 h and lasted in a total of 63 days. The final harvest occurred after nine weeks of flowering (99 DAP) according to previous experiments with the same genotype (Crispim Massuela et al., 2022). Pests were controlled biologically through auxiliary predatory insect populations (*spp. Phytoseiulus persimilis, Amblyseius Californicus*, *Orius Majusculus* and *Aphidoletes aphidimyza*) provided weekly by the company Sautter & Stepper (Ammerbuch, Germany).

### 2.2 Fertilization strategies

#### 2.2.1 Fertilizer type

Two types of liquid fertilizer were used: one organic and a mineral solution. Both fertilizer solutions were mixed and prepared in order to contain the same concentration of macronutrients (N, P, K, Ca and Mg) based on the information provided by the manufacturer. The concentrations [mg L^−1^] of macronutrients in the prepared stock solutions were 240 N, 96 P, 240 K, 18.5 Mg, and 74 Ca (**Table 1**).

**Table 1:** Composition and ratios of fertilizer solutions.

Organic fertilizer solution		Nutrients (mg L^−1^)												Amount added to organic stock solution ^1^
Components	Form	N	P	K	Mg	Ca	S	Fe	Zn	Cu	B	Mo	Mn	
Phytosolution Bio NPK 525	Liquid solution	50000	20000	50000	1300	1300	7000	40	30	4	6	0	16	4.8 (ml)
Carbon Eco	Liquid solution					69000								0.98 (ml)
Epsom salt	Solid salt				12.2		9.9							76.5 (mg)
Organic stock solution (240)		240.0	96.0	240.0	18.5	74.0	43.5	0.19	0.14	0.02	0.03	0.00	0.08	
Organic diluted solution (160) ^2^		160.0	64.0	160.0	12.3	49.4	29.0	0.13	0.10	0.01	0.02	0.00	0.05	
Organic diluted solution (80) ^3^		80.0	32.0	80.0	6.2	24.7	14.5	0.06	0.05	0.01	0.01	0.00	0.03	
Mineral fertilizer solution		Nutrients (mg L^−1^)												
Components	Form	N	P	K	Mg	Ca	S	Fe	Zn	Cu	B	Mo	Mn	Amount added to mineral stock solution ^1^
SSA Ammoniumsulfat	Solid salt	28.5					32.5							135.5 **(**mg**)**
Plantaktiv Typ B	Solid salt	7.8	15.6	23.4	2.0			0.08	0.01	0.03	0.02	0.01	0.04	78 **(**mg**)**
Universol White	Solid salt	123.4		156.3	16.5	74.0		0.82	0.08	0.08	0.08	0.01	0.33	822.5 **(**mg**)**
Wuxal Super	Liquid solution	80.4	80.4	60.3				0.20	0.04	0.04	0.10	0.01	0.12	1005 **(**ml**)**
Mineral stock solution (240)		240.0	96.0	240.0	18.5	74.0	32.5	1.10	0.13	0.15	0.20	0.03	0.49	
Mineral diluted solution (160) ^2^		160.0	64.0	160.0	12.3	49.4	21.7	0.73	0.09	0.10	0.13	0.02	0.33	
Mineral diluted solution (80) ^3^		80.0	32.0	80.0	6.2	24.7	10.8	0.37	0.04	0.05	0.07	0.01	0.16	

1. amount per liter for stock fertilizer solutions (240 mg L^−1^).

2. fertilizer solution 160 was prepared by adding 2 parts of stock solution to 1 part of H_2_O.

3. fertilizer solution 80 was prepared by adding 1 part of stock solution to 2 parts of H_2_O.

The organic fertilizer stock solution was prepared by mixing Phytogreen® Bio NPK 5-2-5 without molasses (Phytosolution, Freyburg, Germany), Carbon Eco (Phytosolution, Freyburg, Germany) and Epsom salt. The proportion and composition of each component are presented in **Table 1**. The mineral fertilizer solution was prepared by mixing commercial horticultural fertilizers, namely: SSA Ammonium sulfate (Raiffeisen AG, Münster, Germany), Plantaktiv Typ B (Hauert, Grossaffoltern, Switzerland), Universol White (ICL Specialty Fertilizers, Ohio, United States) and Wuxal Super (Kwizda Agro GmbH, Vienna, Austria).

The amount of micronutrients provided to plants via substrate and fertilizer was higher than minimum ranges even at the lowest fertilization level (Cockson et al., 2019; Kalinowski et al., 2020). Furthermore, no signs or symptoms of micronutrient deficiencies or toxicities were observed between organic and mineral treatments. Therefore, the nutrient stress object of this study was solely macronutrients.

#### 2.2.2 Fertilizer concentration

Two dilutions were obtained from each the stock fertilizer solution (240). The fertilizer solution 80 and 160 were prepared by adding two parts of stock solution to one or two parts of tap water, respectively. The final concentration for each component is shown in **Table 1**. The treatments (80, 160, 240) refer to fertilizer concentrations in mg L^−1^.

#### 2.2.3 Fertilization sequence

Fertilizer treatments were applied in liquid solution twice per week starting on 22 DAP (**Supplementary Table 2**). The sequence of fertilization events was conceptualized to promote the use of the fertilizer in the substrate and to induce nutrient stress during the flowering period. The volumes were defined based on the plant developmental stage following the fertilizer company recommendations and common practices for indoor cannabis cultivation (Cervantes, 2006). The sequence of fertilization events started at the third week of cultivation with 100 mL plant^−1^ event^−1^ and increased up to 600 mL plant^−1^ event^−1^ in cultivation week ten and eleven. Afterwards, the amount of solution was reduced by 100 mL plant^−1^ event^−1^ until week thirteen. Finally, plants did not receive any further fertilizer during the last week of cultivation. The calculated fertilizer amounts (mg plant^−1^) for N, P and K for each fertilization event and further details on the fertilization scheme can be found in (**Supplementary Table 2**).

A drip irrigation system with a controller was mounted in the pots to provide a constant water supply of 50–500 ml per day depending on the growth stage of the plants and environmental conditions. Water levels in pots were measured by randomized weighing routine and maintained between 40 and 80% of water holding capacity, neither fertilizer solution or irrigation water leached through the pots. All plants received the same amount of fertilizer solution (mL plant^−1^), but nutrient concentrations varied upon fertilizer concentration treatments. The quality of the fertilizer solution was controlled by measuring the pH and EC of the solution at every fertilization event (**Supplementary Table 3**).

### 2.3 Plant sampling

During the cultivation period, two harvests were conducted during the vegetative stage (22 and 36 DAP) and four during the flowering stage (54, 69, 83, and 99 DAP). At each harvest, four plants per treatment (i.e. one plant per treatment and replicate) were cut at the base and separated into three fractions: stems, leaves and inflorescences. At the last two harvests, inflorescences were additionally separated into the main top bud (MTB) and side buds (SB) to account for inner-plant variation in total CBD concentration and CBD yield as demonstrated by Crispim Massuela et al. (2022) and Reichel et al. (2022). Leaf area was measured with an LI-3100 Area Meter (LI-COR, Lincoln, USA), specific leaf area (SLA) (cm^2^ g^−1^) was calculated as the ratio of the leaf area (cm^2^) divided by leaf dry matter (g) for each plant.

At each harvest, SPAD readings were taken at leaves from three different canopy positions based on plant height (low, mid, top). The selected leaves were: for low position the oldest non-senescent fan leaf; for mid position the largest and most developed fan leaf, and for top position the youngest fully developed fan leaf. The average of four measurements were recorded for each leaf with a SPAD 502 (Plus Konica Minolta, Chiyoda, Japan).

### 2.4 Laboratory analysis

Samples of stems and leaves were oven-dried at 60 °C for 48 h. The inflorescences were air-dried in an air-circulated chamber with temperatures ranging between 20–28 °C and relative humidity between 30–60%. All dried samples were weighed to determine dry matter, for cannabinoid analysis, inflorescence samples were ground to homogeneous powder using an ultra-centrifugal mill (Type ZM 200; Retsch, Haan, Germany). The residual moisture of each inflorescence sample was measured with a moisture analyzer (DBS 60-3; Kern and Sohn GmbH, Balingen, Germany).

#### 2.4.1 Cannabinoids

The inflorescence samples were analyzed by high performance liquid chromatography (HPLC), which is the reference method for cannabinoid quantification. The HPLC analysis followed the methods described in Crispim Massuela et al. (2022). The cannabinoid extraction was done using 100 ± 10 mg of ground, dried inflorescences in 100 mL of a methanol 90% and chloroform, 10% (v/v) composite in an ultrasonic bath for 30 min at 40 °C. After cooling down, the solution was filtered through syringe filters Polytetrafluorethylen (PTFE), 0.45 µm (Macherey-Nagel GmbH & Co. KG, Düren, Germany) into HPLC vials and injected into the HPLC system (1290 Infinity II LC System, Agilent, Santa Clara, CA, USA) equipped with an autosampler, a quaternary pump, and a diode-array spectrophotometer (DAD) at the detection wavelength of 230 nm. The chromatographic separation was carried out on a Nucleosil 120-3 C8 column (125 mm × 4 mm i.d., 3.0 µm) with a guard column EC 4/3 Nucleosil 120-3 C8 (Macherey-Nagel, Oensingen, Switzerland). The mobile phase was a mixture of HPLC-grade methanol (solvent A) and 0.1% acetic acid in HPLC-grade distilled H_2_O (solvent B; Sigma-Aldrich, Saint Louis, MO, USA) at a constant flow rate of 0.7 mL min^−1^ with gradient elution mode. The injection volume was 10 µL, with a total run time of 27 min. The integration of targeted peaks was done using cannabinoids analytical reference standards for CBD (C-045) and CBDA (C-144) (Sigma-Aldrich, Darmstadt, Germany) and data analysis was carried out with the software ChemStation for LC Rev. B.04.03-SP2 (Agilent, Santa Clara, CA, USA). Calibration curves were created from diluted standard solutions with a coefficient of determination of 1.0 for both CBD and CBDA. The limit of detection for CBD and CBDA was 0.0015%.

#### 2.4.2 Nutrients

For the nutrient composition analysis of plant material, 0.5 g of each ground dried sample (stems, leaves and inflorescences) was individually weighed and two replicates were pooled to form pooled replicates. For plant samples, total N was measured via Dumas method, total P and total K were measured via ICP-OES. For the substrate nutrient analysis, total N was measured via DIN ISO 11261; 1997-05 and total P and K were measured via DIN EN ISO 11885 (E22); 2009-09.

### 2.5 Statistical analysis

The data was analyzed with a mixed model for all traits to account for the design of the experiment. As pots were arranged in a resolvable row–column design, the model includes block, row and column effects nested within block. The model can be described as:


$$y_{ijklmn}=\mu +b_{l}+r_{lm}+c_{ln}+\alpha_{i}+\beta_{j}+\gamma_{k}+{\left(\alpha \beta \right)}_{ij}+{\left(\alpha \gamma \right)}_{ik}+{\left(\beta \gamma \right)}_{jk}+{\left(\alpha \beta \gamma \right)}_{ijk}+e_{ijklmn}$$

where *y_ijklmn_* is the observation of the plant grown in row *m* and column *n* at table *l* and treated with fertilizer type *i*, fertilizer amount *j* and harvest time *k*, *μ* is the intercept, *b_l_*, *r_lm_*, and *c_ln_* are the random block effects of table *l*, row *m* and column *n* within a table, respectively, *α_i_*, *β_j_*, and *γ_k_* are the fixed main effects of fertilizer type *i*, fertilizer concentration *j* and harvest *k*, respectively. Terms in parenthesis represent fixed interaction effects between the corresponding main effects and *e_ijklmn_* is the error of *y_ijklmn_* with harvest specific variance. Note that fertilizer concentration is a metric variable, so the factor can be replaced by a numeric variable. As there are just three levels, only linear trends can be tested. For most of the variables there was a significant deviation from linearity. Thus, we stick with the factor fertilizer concentration for these variables. For the analysis of nutrient content in plants and inflorescences dry matter/CBD yield, the slope on fertilizer concentration has the interpretation of nutrient uptake efficiency (NUpE) and agronomic nutrient use efficiency (AE), respectively. In both cases, efficiency of nutrients was calculated for fertilizer applied additionally to 80. This requires that the numeric fertilizer amount was coded as 0, 80 and 160 for fertilizer amount 80, 160 and 240, respectively. Therefore, the intercept estimates the mean of treatment 80 and two slopes were fitted for treatments 160 and 240. Note that AE values for N and K were identical, as the amount of N and K was identical within both stock solutions. Further note that the P/N or P/K ratio is also constant. Therefore, tests for nutrient AE were identical, and estimates for N and K were identical, too.

In all cases, normal distribution and homogeneous variances of residuals were checked graphically. If necessary, data were logarithmically transformed prior to analysis. In this case, means and standard errors were back-transformed for presentation purpose only. Standard errors were back-transformed using the delta method. In case of finding significant differences, least square means were compared via Fisher’s LSD test and results were presented via letter display (Piepho et al., 2003).

For the organ inflorescences, data is missing at early harvest resulting in incomplete information of row and column effects. For some traits, this causes convergence problems. To get convergence in these cases, both random effects were dropped from the model. All statistical analyses were conducted by using the software SAS version 9.4 (The SAS Institute, Cary, NC, USA).

### 2.6 Calculations

Total CBD concentration (*CBD_total_* in %) was calculated as the sum of CBD (%) and CBDA (%) in each inflorescence sample. The multiplication by the factor 0.877 accounts for the differences in molar mass between the acid and neutral forms of CBD.


(1) $$CBD_{total}\;=\;CBD\;+\;CBDA\times 0.877$$

Yield of total CBD (*CBD_yield_* in mg plant^−1^) was calculated considering inflorescence dry matter (*DM_flo_*), the residual moisture (*RM*) of the analytical sample and the *CBD_total_*. The *RM* was the weight proportion of water in the analytical dried samples (ranged between 0.0663 and 0.0936). The CBD*_yield_* was calculated at zero moisture content for each sample as follows:


(2) $$CBD_{yield}=\left(CBD_{total}-RM\right)\times DM_{flo}=\left(CBD_{total}\times DM_{flo}\;\right)-\left(RM\times DM_{flo}\right)$$

Based on the nutrient concentration and the plant organ dry matter, the nutrient content was calculated for each individual organ – stems, leaves and inflorescence – and their sum was denoted as plant nutrient content (*Nutrient content_plant_*). The sum of plant dry matter is denoted as (*DM_plant_*)

Then, the Nutrient Utilization Efficiency (NUtE) and the Biomass and Nutrient Harvest Index (HI) ratios were calculated as follows:


(3) $$Nutrient\;Utilization\;Efficiency\;\left(NUtE\right)=\frac{DM_{flo}}{Nutrient\;content_{plant}}$$

(4) $$Biomass\;harvest\;index=\;\frac{DM_{flo}}{DM_{plant}}$$

(5) $$Nutrient\;harvest\;index=\;\frac{Nutrient\;content_{flo}}{Nutrient\;content_{plant}}$$

## 3 Results

**Figure 1:**
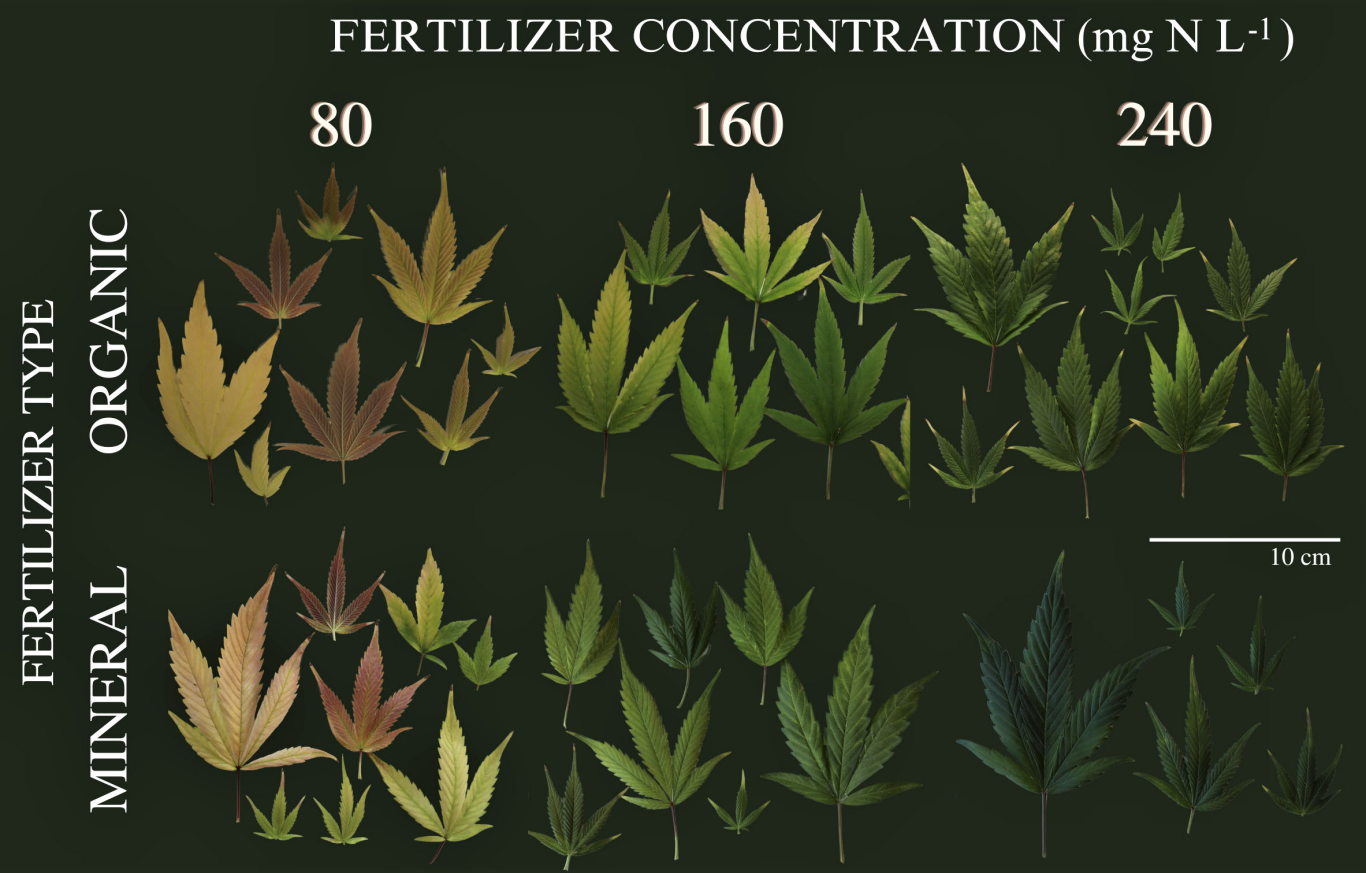
Leaves from cannabis plants of the last harvest at 99 days after planting. Leaves are from different canopy positions and illustrated for the factors fertilizer type (vertical) and fertilizer concentration (horizontal).

During the vegetative period, no visual symptoms of nutrient deficiency were observed for the six treatments as illustrated in (**Supplementary Figure 1**) for exemplary plants during the second harvest event at the end of vegetative phase (36 DAP). This indication by visual appearance was further confirmed by the absence of significant differences between treatments for the plant variables measured during the vegetative phase as shown in the following. In contrast, as illustrated in **Figure 1**, leaf coloration showed obvious symptoms of nutrient deprivation by the last harvest (99 DAP) in leaves for both organic and mineral fertilizer types, which in general became more pronounced with decreasing fertilizer concentration.

### 3.1 Interactions among factors fertilizer type and concentration

The results are divided into six sub-sections based on the main factors fertilizer type and fertilizer concentration for the variables observed over time: biomass and cannabinoid accumulation, nutrient allocation, translocation and re-mobilization among the plant organs, and nutrient use efficiencies.

As presented in **Table 2**, the interaction of the factors fertilizer type and fertilizer concentration were not significant for plant and inflorescence dry matter, leaf area and specific leaf area, CBD concentration and yield. Therefore, marginal means were presented separately in the following sub-sections.

**Table 2:** ANOVA table for the sources of variation Fertilizer (Fert) Type, Fertilizer Concentration (Conc), Days after planting (DAP), and their interactions.

Sources of variation	Plant dry matter	Leaf Area	SLA	Inflorescence dry matter	Total CBD%	CBD Yield
**Fert Type**	0.0015	**0.0061**	0.768	<.0001	0.0183	**0.0173**
**Fert Conc**	<.0001	<.0001	0.0004	<.0001	**0.0279**	<.0001
Fert Type*Fert Conc	0.7009	0.8994	0.6353	0.5988	0.6832	0.9428
**DAP**	<.0001	<.0001	<.0001	<.0001	<.0001	<.0001
DAP*Fert Type	**0.0313**	0.4664	0.2219	**0.0125**	**0.0451**	0.4105
DAP*Fert Conc	**<.0001**	**<.0001**	**0.0297**	**<.0001**	0.5256	**0.0239**
DAP*Fert Type*Fert Conc	0.0580	0.2163	0.5655	0.2400	0.5382	0.315

*p*-Values correspond to the F-test for differences between levels of the corresponding factor. The *p*-values with statistical significance (α=0.05) were highlighted in bold to facilitate the visual representation.

### 3.2 Fertilizer type

**Figure 2:**
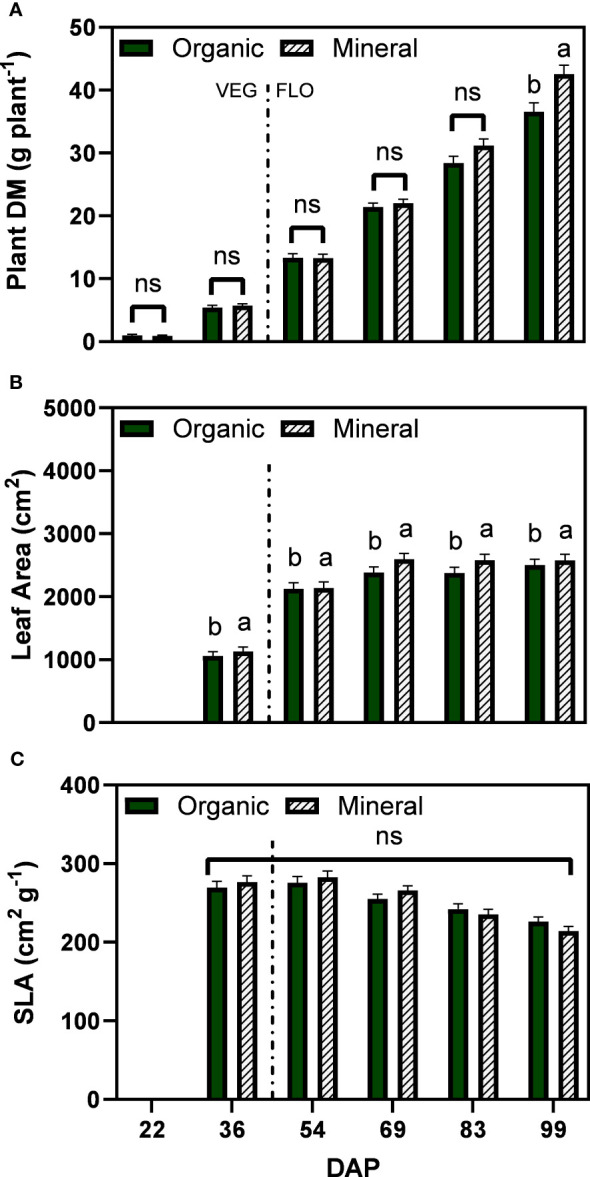
Least square means (±standard error) of **(A)** plant dry matter (DM), **(B)** plant leaf area, and **(C)** specific leaf area (SLA) for the organic and mineral fertilizer treatment and six harvest dates (Days after planting, DAP). For each harvest date, means with at least one identical letter are not significant different from each other according to Fisher’s LSD test with α=0.05. Values with (ns) are not significantly different from each other according to global F-test. The dashed line marks the conversion of the vegetative (VEG) to flowering (FLO) stage after 36 DAP. For **(B)**, letters are based on marginal mean comparisons and were repeatedly presented for each DAP.

Plant dry matter increased steadily over time for both fertilizer types and showed marginal differences between 22 and 69 DAP (**Figure 2A**). At the last two harvests differences became more pronounced with significant differences between fertilizer types at the last harvest date (99 DAP), reaching 36.6 g plant^−1^ for the organic and 42.5 g plant^−1^ for the mineral fertilizer treatments. For the leaf area per plant, the interaction of fertilizer type and DAP was not significant, however, fertilizer type was significant with larger leaf area for the mineral treatments (2205 cm²) compared with the organic treatments (2090 cm²) (**Figure 2B**). No significant differences between fertilizer types were found for specific leaf area, which in general showed a decreasing trend from 69 DAP and ranged between 282.3 to 214.2 cm² g^−1^ (**Figure 2C**).

**Figure 3:**
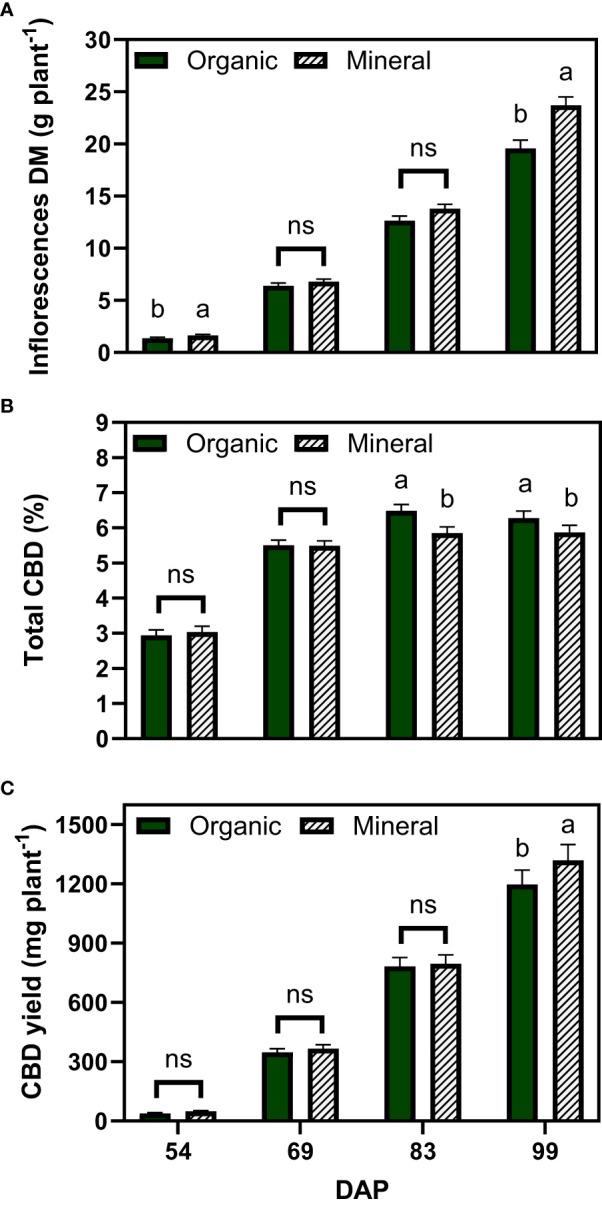
Least square means (±standard error) of **(A)** inflorescence dry matter (DM), **(B)** total CBD concentration, and **(C)** total CBD yield at zero moisture for the organic and mineral fertilizer treatments and four harvest dates (Days after planting, DAP). For each harvest date, means with at least one identical letter are not significant different from each other according to Fisher’s LSD test with α=0.05. Values with (ns) are not significantly different from each other according to global F-test.

The inflorescence dry matter also increased over time and significant differences were found at the last harvest date, with an average of 19.6 g plant^−1^ for the organic and 23.7 g plant^−1^ for the mineral fertilizer treatments (**Figure 3A**). Inflorescence dry matter made up a large proportion of the plant dry matter with a biomass harvest index of 53.5% for organic and 55.7% for the mineral fertilizer at the last harvest (not shown).

The total CBD concentration of inflorescences increased from 2.9% to 6.5% for organic and from 3.0% to 5.8% for mineral fertilizer treatments, measured 45 and 83 DAP, respectively (**Figure 3B**). Total CBD concentration did not change significantly between the last two harvests. Contrary to the results for inflorescence dry matter, organic fertilization significantly increased CBD concentrations by 12% compared with mineral fertilization.

Total CBD yield was calculated at zero moisture with the multiplication of inflorescence dry matter and total CBD concentration as described in equation 2. Following the plant dry matter accumulation trends, results among fertilizer types were significantly different only for the final harvest date, whereas mineral fertilizer treatments accumulated an average of 1319 mg CBD plant^−1^ in comparison with 1197 mg CBD plant^−1^ for the organic fertilizer treatments.

### 3.3 Fertilizer concentration

**Figure 4:**
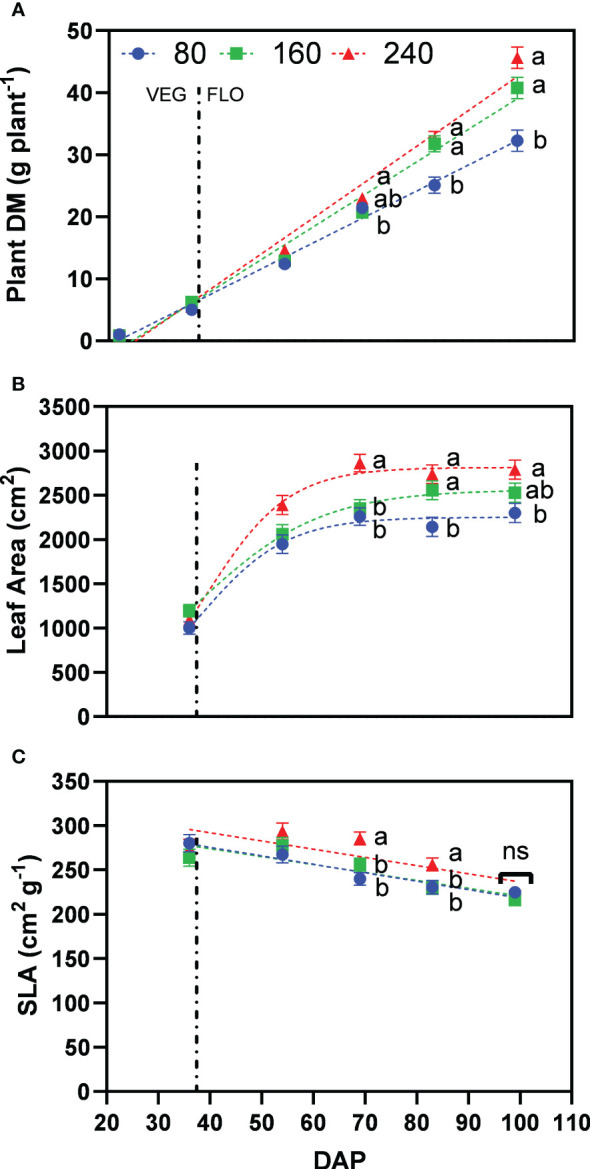
Least square means (±standard error) of **(A)** plant dry matter (DM), **(B)** plant leaf area, and **(C)** specific leaf area (SLA) for the fertilizer concentration treatments (80, 160 and 240 mg N L^−1^) at different harvest dates (Days after planting, DAP). For each harvest date, means with at least one identical letter are not significant different from each other according to Fisher’s LSD test with α=0.05. Values with (ns) are not significantly different from each other according to global F-test. The vertical line marks the conversion of the vegetative (VEG) to flowering (FLO) stage after 36 DAP. Dash lines are trendlines used only for visual representation.

Increasing fertilizer concentrations resulted in a higher plant dry matter with 32.3, 40.8, and 45.6 g plant^−1^ for the treatments 80, 160 and 240, at the last harvest date, respectively (**Figure 4A**). Differences between 160 and 240 were not significant. Leaf area also increased with higher fertilizer concentrations, with 2299, 2529 and 2791 cm² plant^−1^ for the treatments 80, 160 and 240 at the last harvest date, respectively (**Figure 4B**). Differences between 160 and 240 were not significant. A significantly larger leaf area was observed for 240 at 69 and 83 DAP as a result of a significantly higher specific leaf area, but being not significantly different at the last harvest (99 DAP) (**Figure 4C**).

**Figure 5:**
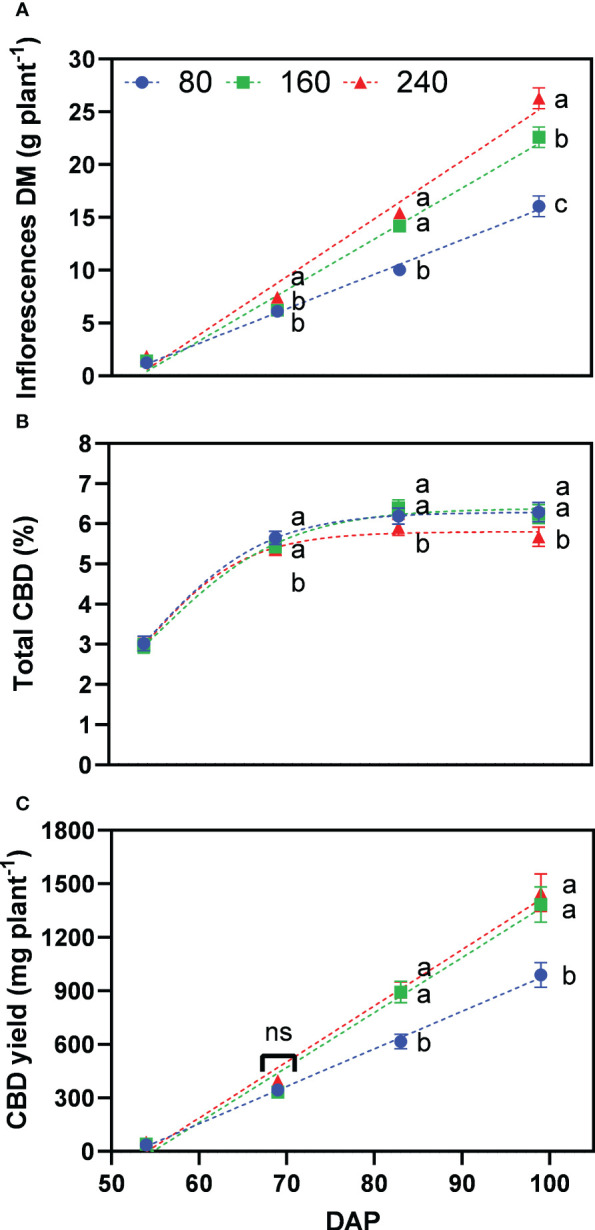
Least square means (±standard error) of **(A)** inflorescence dry matter (DM), **(B)** total CBD concentration, and **(C)** total CBD yield at zero moisture for the fertilizer concentration treatments (80, 160 and 240 mg N L^−1^) at different harvest dates (Days after planting, DAP). For each harvest date, means with at least one identical letter are not significant different from each other according to Fisher’s LSD test with α=0.05. Values with (ns) are not significantly different from each other according to global F-test. Dash lines are trendlines used only for visual representation. For **(B)**, letters are based on marginal mean comparisons and were repeatedly presented for each DAP.

Inflorescence dry matter accumulation increased steadily during the whole flowering period, with 16.1, 22.6, and 26.3 g plant^−1^ for the treatments 80, 160 and 240, at the last harvest date, respectively (**Figure 5A**). Significant differences between treatments 160 and 240 only occurred in the last weeks of flower development, being the major sink at this stage. At the initiation of flowering (69 DAP), treatment 80 was not significantly different than 160, and both were significantly lower than the well-fertilized treatment 240.

While inflorescence dry matter increased with fertilizer concentration, total CBD concentration across harvest dates was significantly lower at 240 with 4.98% compared with 5.36% for 160 and 5.29% for 80, respectively – the latter two were not significantly different from each other (**Figure 5B**). The interaction between fertilizer concentration and DAP was not significant. At the final harvest, plants of treatments 80, 160 and 240 produced 6.3%, 6.2% and 5.7% of total CBD concentration, respectively.

Following the differences for inflorescence dry matter, the lowest fertilizer concentration resulted in significantly lower total CBD yield with 989 mg plant^−1^ for 80 compared to 1384 and 1450 mg plant^−1^ for 160 and 240, respectively, which were not significantly different from each other (**Figure 5C**). Thus, the fertilizer concentration 160 produced 95% of the CBD yield of 240, while receiving one-third less nutrients.

### 3.4 Nutrients re-mobilization

**Figure 6:**
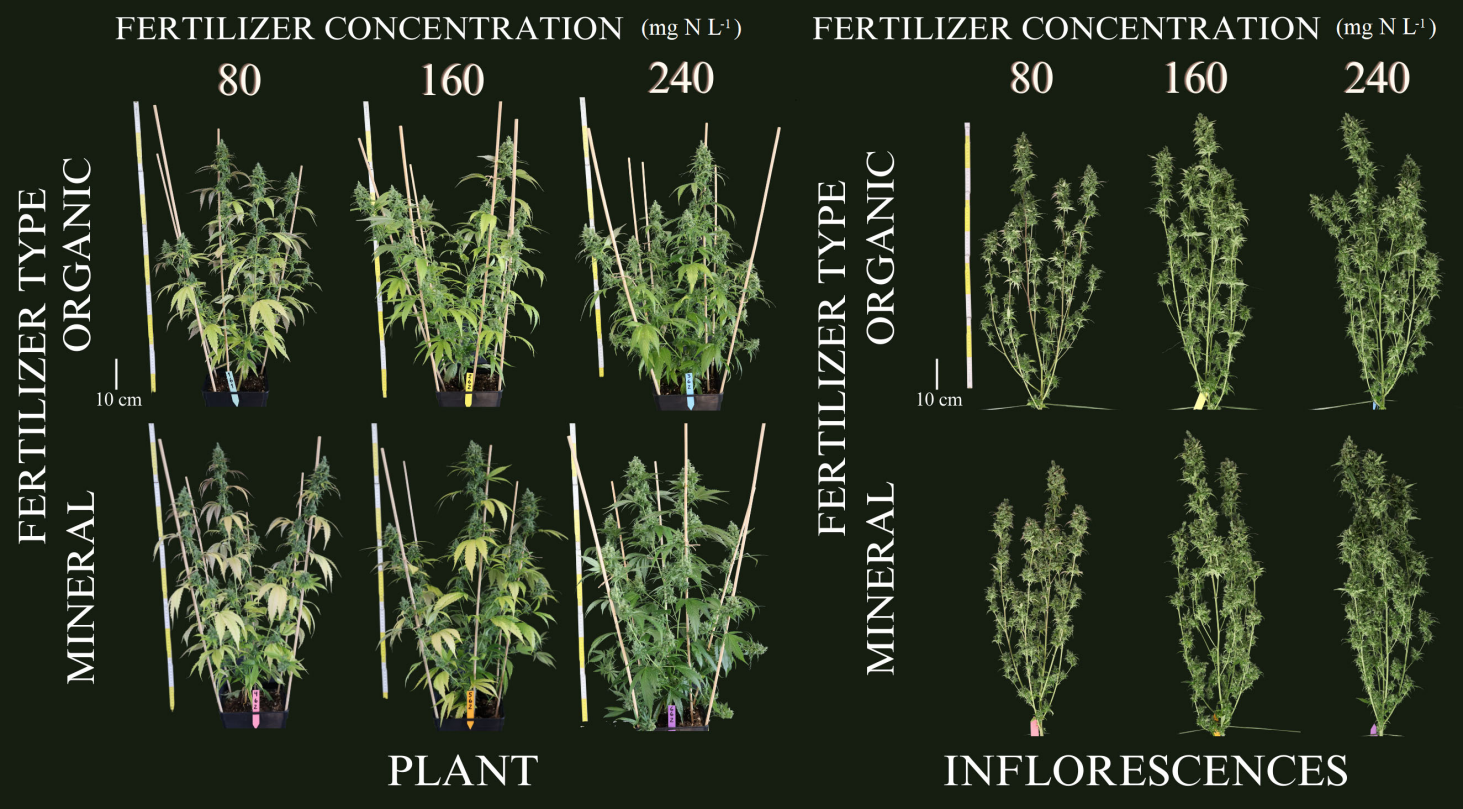
Cannabis plants at the final harvest date (99 days after planting) for the three organic and mineral fertilizer concentrations. Left: entire plants, Right: defoliated plants.

During growth, plants take up and utilize nutrients in different plant organs. Under nutrient deprivation, older (fan) leaves translocate nutrients to younger (sugar) leaves and inflorescences. During inflorescence growth, nutrients are re-mobilized and thus, the green pigmentation in inflorescences is maintained, while the pigmentation of leaves changes as illustrated in **Figures 1** and **6**.

The statistical analysis did not show any significant interactions between fertilizer type and concentration but interactions of both factors with DAP except for N content (**Tables 3**, **4**). For the latter, the *p*-value of the three-way interaction was close to 0.05. Additionally, F values for two-way interactions were large and visual inspection showed no crossing interactions. Therefore, the data is presented separately for each DAP for all variables. Additionally, results were presented for the effect of fertilizer concentration only, as it was considered the most relevant factor for the nutrient re-mobilization among plant organs.

**Table 3:** ANOVA table for the Effects Fertilizer (Fert) Type, Fertilizer Concentration (Conc), Days after planting (DAP), and their interactions.

Organ	ANOVA Table						
	Source of variation	N Conc.	P Conc.	K Conc.	N Content	P Content	K Content
**Leaves**	**FertType**	0.0235	0.7612	0.0188	<.0001	0.044	<.0001
	**FertConc**	<.0001	<.0001	<.0001	<.0001	<.0001	<.0001
	FertType*FertConc	0.8428	0.3772	0.886	0.981	0.4972	0.8839
	**DAP**	<.0001	<.0001	<.0001	<.0001	<.0001	<.0001
	DAP*FertType	**0.0212**	**0.0056**	**0.0319**	**0.0002**	**0.0381**	**0.0023**
	DAP*FertConc	**0.0011**	**0.0298**	**0.0003**	**<.0001**	**<.0001**	**<.0001**
	DAP*FertType*FertConc	0.3339	0.1627	0.3187	0.1554	0.2032	0.4609

*p*-Values correspond to the F-test for differences between levels of the corresponding factor on concentration and content of N, P, and K in leaves. The *p*-values with statistical significance (α=0.05) were highlighted in bold to facilitate the visual representation.

**Table 4:** ANOVA table for the Effects Fertilizer (Fert) Type, Fertilizer Concentration (Conc), Days after planting (DAP), and their interactions.

Organ	ANOVA Table						
	Source of variation	N Conc.	P Conc.	K Conc.	N Content	P Content	K Content
**Inflorescences**	**FertType**	**0.0023**	0.0074	0.0002	<.0001	0.0002	<.0001
	**FertConc**	<.0001	0.0004	**<.0001**	<.0001	<.0001	<.0001
	FertType*FertConc	0.3495	0.3295	0.2462	0.3463	0.4993	0.2721
	**DAP**	0.0262	<.0001	<.0001	<.0001	<.0001	<.0001
	DAP*FertType	0.1581	**0.0056**	**0.0054**	0.0009	**0.0101**	**0.0019**
	DAP*FertConc	**0.0368**	**0.0221**	0.0612	<.0001	**<.0001**	**<.0001**
	DAP*FertType*FertConc	0.624	0.9796	0.3673	**0.0364**	0.0827	0.0518

*p*-Values correspond to the F-test for differences between levels of the corresponding factor on concentration and content of N, P, and K in inflorescences. The *p*-values with statistical significance (α=0.05) were highlighted in bold to facilitate the visual representation.

**Figure 7:**
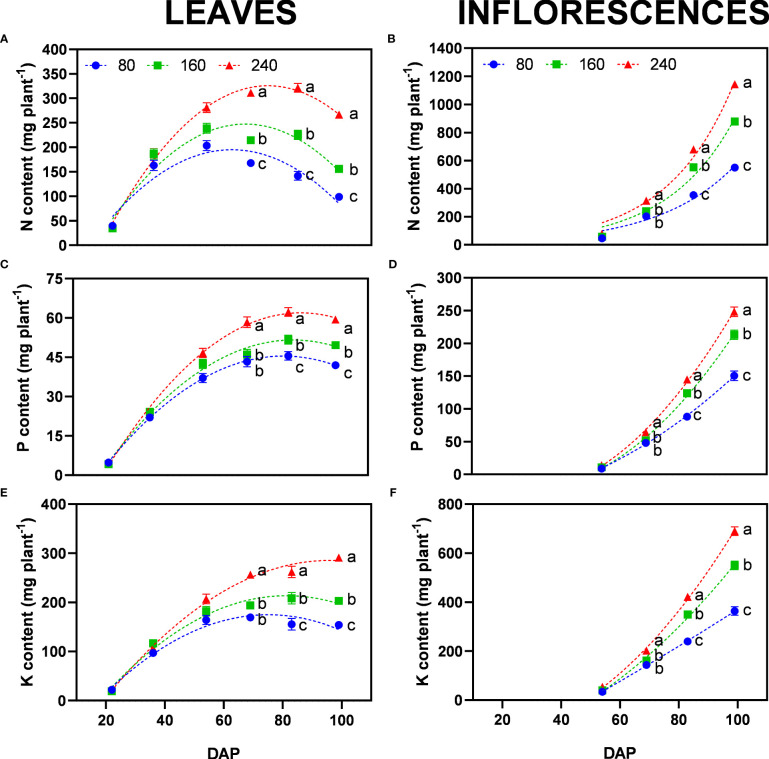
Least square means (±standard error) for the content of **(A, B)** nitrogen, **(C, D)** phosphorus, and **(E, F)** potassium in leaves (left) and inflorescences (right) for the fertilizer concentrations (80, 160 and 240 mg N L^−1^) at different harvest dates (days after planting, DAP). For each harvest date within each single figure, means with different letters were significantly different from each other according to Fisher’s LSD test with α=0.05.

The highest fertilizer concentration (240) can be considered as the well-fertilized control, as no nutrient deficiency symptoms were observed and both leaves and inflorescences constantly accumulated nutrients (**Figure 7**).

Nitrogen is easily mobile in plant tissue, which can be observed by the presented curves of accumulation and depletion of nitrogen content in leaves (**Figure 7A**). The rate of remobilization based on different fertilizer concentrations was apparent for nitrogen-deprived plants (80 and 160). Plants of these treatments reached their peak N content in leaves at 54 DAP and then decreased as N was re-mobilized and translocated to inflorescences, the largest sink at this stage (**Figures 7A, B**). Since leaf dry matter was not reduced over time (**Figure 4**), the major changes appeared at the chemical level, generally reducing leaf N concentration (**Supplementary Table 4**). The reduction in N concentration was also present for the control treatments (240) indicating a preference of the plant to shift N to inflorescences, however, the beginning of re-mobilization occurred later as indicated by the peak N-content in leaves reached at 83 DAP (**Figure 7A**).

Phosphorus is also a mobile nutrient in plants. However, P mobilization from leaves to inflorescences was not clearly visible like for N. All treatments reached a plateau for P content in leaves at 69 DAP (**Figure 7C**). At the final harvest, plants of 80, 160 and 240 accumulated 41.9, 49.7, and 59.4 mg P plant^−1^ in the leaves, respectively. P content in inflorescences was up to 4.2 times higher than in leaves (**Figure 7D**). Differences between fertilizer concentrations were significant with 151, 213, and 248 mg P plant^−1^ in the inflorescences of the 80, 160 and 240 treatments, respectively. Interestingly, in all treatments, the concentration of P in inflorescences was not significantly different at the last harvest, indicating a maximum chemical accumulation of P in floral tissue independent of the amount of fertilizer provided (**Supplementary Table 4**).

The accumulation dynamics of K were similar to P with the only exception that plants for the lowest fertilizer concentration (80) reached their plateau earlier at 69 DAP (**Figure 7E**). Well-fertilized treatments accumulated K until the last harvest, possibly correlated with the higher plant biomass as K was mostly stored in the stem, at the initiation of growth.

### 3.5 Nutrient translocation

**Figure 8:**
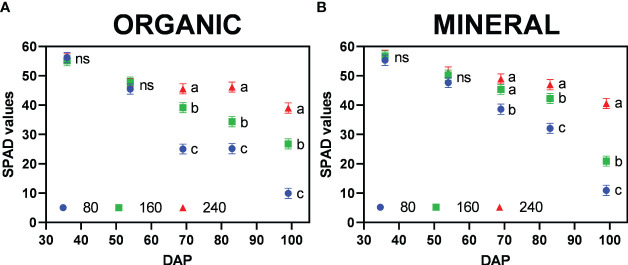
Mean SPAD values per plant (±standard error) for the fertilizer concentration treatments (80, 160 and 240 mg N L^−1^) for **(A)** organic and **(B)** mineral fertilizer type at different harvest dates (Days after planting, DAP). Symbols represent fertilizer concentrations: dots = 80, squares = 160, and triangles = 240. For each fertilizer type and harvest date, means with different letters are significantly different from each other according to Fisher’s LSD test with α=0.05. Values with (ns) are not significantly different from each other according to global F-test.

At the end of the vegetative period (36 DAP) all treatments indicated SPAD values above 50 with no significant differences between them (**Figure 8**). After four weeks in the flowering period (> 64 DAP), a general decreasing trend in SPAD values became apparent, in particular for the treatments with a moderate (160) and severe nutrient stress (80) indicating differences in N re-mobilization from leaves. For the organic treatments a linear decrease was observed, whereas for mineral fertilizer treatments, a considerable reduction of 49.5% in SPAD values occurred between the last two harvests for 160, a trend which was also observed for the lowest fertilizer concentration (80). Obtained results indicate a higher nutrient demand of inflorescences at the end of the flowering period (between seven and nine weeks post-anthesis), when inflorescence dry matter increased most, thus being a strong sink.

SPAD values for each harvest and leaf position are presented in **Supplementary Table 5**. Trends such as the higher reduction (%) of SPAD for lower fertilizer treatments depended on leaf position, suggesting the vertical movement of N from the oldest (low) to the youngest (top) leaves, where higher values were sustained until the last harvest. At the highest nutrient deficiency (80) nutrients were mobilized from leaves of all positions. In the 240 treatments, plants remained green and maintained SPAD values above 40, showing no nutrient deficiencies. Burgel et al. (2020) also reported ranges of SPAD values between 40 to 60 for healthy green cannabis leaves. The trends are confirmed by chemical analysis of total plant nutrient concentration (**Supplementary Table 4**). Leaf chlorosis could be observed by SPAD values between 40 to 20 and in particular a change in leaf color from green to yellow. These values have also been previously reported (Mayer et al., 2015). Significant differences in SPAD values were only observed after 60 DAP, indicating initiation of nutrient depletion at the mid-flowering stage (by the fourth week, post-anthesis). Below SPAD values of 20, leaves would appear pale yellow and whiteish (indicating a complete lack of chlorophyll) and red/purple colors (anthocyanin expression) by SPAD values below 10, as represented in **Figure 1**.

### 3.6 Nutrient use efficiency

Five nutrient efficiency indexes were calculated as presented in Sections 2.5 and 2.6. The indexes cover the nutrient dynamics from substrate to plant (NUpE); the utilization of nutrients in plant to produce inflorescences biomass (NUtE); as the agronomic efficiency related to fertilizer use (AE) for inflorescences and CBD yield and the efficiency in harvesting nutrients from the system (N-HI).

The inflorescence nutrient harvest index (N-HI) was significantly different only for P, where mineral treatments harvested in inflorescences 76% of the P content in the plant compared to 71% of the organic treatments. For N and K results are not significantly different, showing the same capacity of plants to allocate N and K in the inflorescences across fertilizer types and between fertilizer treatments 160 and 240 (**Table 5**). This trend is also confirmed by the nutrient utilization efficiency, where P-NUtE was significantly higher for mineral treatments (81) than for organic treatments (75), but results were not significantly different for N and K. Additionally, P utilization was 4 times higher than N utilization, indicating the importance of P to build inflorescence biomass (**Table 5**).

**Table 5:** Least square means (±standard error) and *p*-values for nutrient harvest index (N-HI) and nutrient utilization efficiency (NUtE) for the factors fertilizer type (FertType) and fertilizer concentration (FertConc) treatments.

Index	Nutrient	LS Means ± SE				*p*-values	
		Fertilizer Type		Fertilizer Concentration			
		Organic	Mineral	160	240	FertType	FertConc
**N-HI**	**N**	0.79 ± 0.01	0.8 ± 0.01	0.81 ± 0.01	0.78 ± 0.01	0.5343	0.1962
	**P**	0.71 ± 0.005 **b**	0.76 ± 0.005 **a**	0.74 ± 0.01	0.74 ± 0.01	**0.001**	0.2203
	**K**	0.64 ± 0.01	0.65 ± 0.01	0.67 ± 0.01	0.65 ± 0.01	0.456	0.0942
**NUtE**	**N**	20.74 ± 0.37	20.53 ± 0.37	20.79 ± 0.45 **a**	17.82 ± 0.45 **b**	0.7074	**0.0011**
	**P**	75.05 ± 0.84 **b**	81.01 ± 0.84 **a**	78.94 ± 1.02	77.89 ± 1.02	**0.0039**	0.546
	**K**	26.57 ± 0.4	26.88 ± 0.4	27.61 ± 0.49 **a**	24.71 ± 0.49 **b**	0.6117	**0.0106**

Means with different letters are significantly different from each other according to Fisher’s LSD test with α=0.05. Means without letters are not significantly different from each other according to global F-test. The *p*-values with statistical significance (a=0.05) were highlighted in bold to facilitate the visual representation.

Moreover, under nutrient stress, the efficiency in allocating and utilizing plant N and K to build inflorescences dry matter (NUtE) increased significantly in the treatment 160 in comparison to 240 for N (20.8 compared with 17.8) and for K (27.6 compared with 24.7), respectively (**Table 5**).

Generally, figures show higher nutrient uptake and agronomic use efficiencies for mineral in comparison to organic treatments. Besides that, a more efficient use of fertilizers is seen when plants have nutrient stress (for the treatment 160 in comparison to 240). Differences between fertilizer concentrations are also more pronounced in mineral treatments, reinforcing the higher availability – and thus higher nutrient uptake – of nutrient forms in mineral than organic fertilizer.

The significantly lower plant and inflorescence dry matter, leaf area and finally CBD yield of organic treatments (**Figures 2**, **3**) are supported by the general lower nitrogen uptake efficiency than mineral treatments (**Table 6**), which resulted in a lower amount of N available to plant’s growth, especially in the major sink stage (last two weeks of the experiment, as described in the prior sections). Finally, this is transcribed by the much higher agronomic use efficiency (AE*_inflorescences_*) of mineral fertilizers, being twice as higher in comparison to organic for the 160 treatments.

**Table 6:** Least square means (±standard error) and *p*-values for nutrient uptake efficiency (NUpE) and nutrient agronomic use efficiency for inflorescence dry matter (AE_inflorescences_) and for CBD yield (AE_CBDyield_) for the interaction of factors fertilizer type (FertType) and fertilizer concentration (FertConc).

Index	Nutrient	Slope estimates ± SE				*p*-values
		Fertilizer Type				Ntfertilizer* FertType* FertConc
		Organic		Mineral		
		Fertilizer Concentration				
		160	240	160	240	
**NUpE**	**N**	0.55 ± 0.08	0.58 ± 0.04	0.74 ± 0.08	0.71 ± 0.04	0.5116
	**P**	0.25 ± 0.08	0.23 ± 0.04	0.39 ± 0.08	0.3 ± 0.04	0.501
	**K**	0.29 ± 0.06	0.35 ± 0.03	0.5 ± 0.06	0.45 ± 0.03	0.2101
**AE_inflorescences_**	**N/K**	7.22 ± 2.41	7.1 ± 1.2	14.25 ± 2.41	9.74 ± 1.2	0.1967
	**P**	18.05 ± 6.02	17.75 ± 3.01	35.62 ± 6.02	24.34 ± 3.01	
**AE_CBDyield_**	**N/K**	0.47 ± 0.24	0.35 ± 0.12	0.76 ± 0.24	0.44 ± 0.12	0.521
	**P**	1.18 ± 0.61	0.88 ± 0.3	1.9 ± 0.61	1.1 ± 0.3	

Results show low uptake of P in both fertilizer types in comparison to N uptake. For organic fertilizer only around 24% of the P provided to the plant was actually taken up (**Table 6**), indicating low availability of P in the substrate and fertilizer solution, which is generally the case for peat substrates, where P is easily bound (Schachtman et al., 1998). For mineral fertilizer, P uptake was higher; 30% for 240 and 39% for 160, indicating higher availability of P in mineral forms from fertilizer, which could also have influenced the higher mobility and allocation of P in inflorescences, resulting also in higher NUtE (**Tables 5**, **6**). Accordingly, for mineral treatments under nutrient stress (160) the agronomic use efficiency of P increased by 46% for inflorescence dry matter and 72% for CBD yield production (**Table 6**). This supports observations on the higher efficiency of re-mobilizing P among plant organs (**Figure 7**) due to limited P uptake (**Table 6**), which remained in the substrate (**Supplementary Table 4**).

Finally, plants under nutrient stress (160) could increase their agronomic nutrient use efficiency to produce CBD output in comparison to well-fertilized plants (240). AE*_CBDyield_* for N and K increased 34% for the organic fertilizers (an increase of 0.47 to 0.35) and 72% for the mineral fertilizers (an increase of 0.76 to 0.44)

The results for K follow the trends presented for N, as both were provided in the same ratio and quantity. However, K had a lower uptake efficiency and harvest index in comparison to N, as K assimilation would compete with other cations like Ca^+^ and Mg^+^. That is confirmed by the values of K uptake efficiency for mineral fertilizer, as additional K inputs were not taken up by plants. Nonetheless, this is not true for the organic treatments.

## 4 Discussion

This is the first study showing the potential to reduce fertilizer input while maintaining CBD yield of medicinal cannabis. Even though inflorescence yield was lower at the final harvest, this was compensated by a higher CBD concentration, a trend found across fertilizer types. Furthermore, we found that the higher nutrient use efficiency of N, P, and K was achieved by a larger mobilization and translocation of nutrients, increasing the utilization efficiency of acquired nutrients. Differences in CBD yield between fertilizer types occurred only at the final harvest, where the higher CBD concentration could not compensate for the lower inflorescence dry matter. Our results showed a lower acquisition and utilization efficiency for the organic fertilizer. There were no significant interactions between fertilizer type and concentration for the analyzed variables: yield, nutrient concentration, content and efficiency indices. This study contributes to the growing body of scientific evidence that fertilizer use efficiency can be improved in the cultivation of medicinal cannabis with the aim to reduce negative environmental impacts related to the excessive use of fertilizers.

### 4.1 Does fertilizer type matter?

Several mineral and organic liquid fertilizer solutions are commercially available. Previous studies showed differences in inflorescence biomass, CBD yield and nutrient use efficiency for organic and mineral fertilizers. Our results partly confirm the need for higher concentrations of organic fertilizers to reach the productivity of mineral fertilizers, as indicated by optimum nutrient concentrations for organic of 212–261 mg N L^−1^ (Caplan et al., 2017a) in comparison to 160 mg N L^−1^ for mineral fertilizers (Saloner and Bernstein, 2021) due to the lower nutrient uptake efficiency.

Our study revealed, in addition, that the difference in final yield was mainly attributed to the last two weeks before harvest, when the mineral fertilization had significantly higher inflorescence dry matter than organic treatments (**Figure 3**). This was supported by the higher nutrient use efficiencies of mineral nutrients. However, differences in CBD yield between fertilizer types were lower due to lower CBD concentration with mineral fertilization (**Figure 3**), indicating a possible dilution effect (Shiponi and Bernstein, 2021b; Bruce et al., 2022). Vegetative plant growth was not affected by the type of fertilizer as indicated by similar values of leaf area and specific leaf area (**Figure 2**). During the last two weeks before harvest, inflorescence dry matter strongly increased and cannabinoids were accumulated, presenting a large sink with high assimilate and nutrient demand (**Figure 3**). During this phase, plants receiving mineral fertilizer maintained a faster growth indicating that the mineral form of nutrients was more readily available for uptake compared with the organic nutrients.

The allocation of mineral nutrients between plant organs (**Figure 6**) showed the translocation of individual macro elements in relation to plant organs and age as also reported in other studies for medicinal cannabis (Bernstein et al., 2019; Malík et al., 2021). Nutrient stress levels were comparable in both fertilizer types as indicated by similar SPAD values (**Figure 8**). The highest translocation rate of nitrogen (i.e., reduction of SPAD value) occurred also during the last two weeks before harvest – especially for mineral fertilizers – indicating that plants could not provide nutrients for inflorescence growth mainly by root uptake, being the re-mobilization of nutrients necessary at that point. For organic fertilizers, the re-mobilization occurred before that, as the nutrient uptake efficiency (NUpE) was lower, and thus the acquisition of nutrients throughout the flowering period.

The lower NUpE of organic fertilizers is in general related to the complexity of biological interactions that are necessary to convert and make nutrients available. Several microorganisms like fungi and bacteria are responsible to mineralize organic nutrients and improve their availability to plants (Lowenfels and Lewis, 2010; Lowenfels, 2013). One main difference among nitrogen forms in fertilizer types is that organic fertilizers contain higher ratios of NH_4_^+^ to NO_3_^−^ than mineral fertilizers.

The impact of N form on cannabis plant function and production was also demonstrated by a 46% decrease in inflorescence yield with the increase in the share of N supplied as NH_4_^+^ from 0 to 50% (Saloner and Bernstein, 2022). Yet, moderate levels of 10–30% of NH_4_^+^ showed only minor adverse effects on plant function and secondary metabolism but produced lower inflorescence yields compared with pure NO_3_^−^ nutrition. Under a level of 50% NH_4_^+^, the plants demonstrated toxicity symptoms, which impaired plant growth. In our study, NH_4_^+^ toxicity symptoms were also observed in plants of the highest organic fertilizer treatment (240), which showed burned tips of leaves (**Figure 1**). In an outdoor experiment by Bruce et al. (2022), commercial organic solid fertilizer increased inflorescence biomass compared to mineral fertilizer treatments, while the concentration of cannabinoids was not altered. The highest CBD concentrations were found for manure-based compost treatments, which produced the lowest inflorescence yield in the first year, but the highest in the second year. This indicates a time interaction between nutrient availability and the ratios of NH_4_^+^ to NO_3_^−^ (Bergstrand et al., 2019).

Besides yield metrics, organic fertilizers could enhance quality traits, e.g. a higher lycopene concentration in tomatoes compared to mineral fertilizers was reported (Bilalis et al., 2018). Authors reported a non-significant difference in lycopene yield between organic and conventional tomatoes, suggesting organic production as a suitable alternative for lycopene production. Long-term studies demonstrated that NUE increased when multi-nutrient and organic fertilizers were used in field conditions (Zhu et al., 2023), suggesting a limited interpretation of comparative results between higher efficiencies of mineral to organic fertilization strategies. This can be true for single-use substrates but seems not to be the case in other cultivation systems. It can be observed that organic medicinal cannabis cultivation facilities are adopting raised beds and living soil systems that are used for several cultivation batches.

Micronutrients (Cu, Fe, Mn, and Zn) are important co-factors of the group of enzymes superoxide dismutases that detoxifies reactive oxygen species, which normally accumulate more under both biotic and abiotic stress conditions than under ambient conditions. B is important in cell wall formation and flowering. Apart from Zn, these micronutrients were present in higher concentrations in the mineral fertilization regimes than in the organic. This may have influenced the results. However, as pointed out in section 2.2.1, we assume this effect negligible as no deficiency or toxicity symptoms were observed during the experiment.

### 4.2 Can fertilizer concentration be reduced?

The reported environmental impact and carbon footprint (Mills, 2012) associated with the fast increase of commercial cannabis cultivation draw attention toward a more efficient use of fertilizers (Wartenberg et al., 2021). The response of plant growth to an increase in fertilizer concentration often follows a convex bell-shaped curve. As demonstrated, cannabis growth responds positively until an optimum amount of N, P and K and then decreases at higher rates (Bevan et al., 2021).

Our results showed that plant growth responded positively from the lowest to the highest fertilizer concentration with an increase in plant and inflorescence dry matter and leaf area (**Figures 4** and **5**), as confirmed by other studies in literature (Bevan et al., 2021; Malík et al., 2021). The final CBD yield, however, did not show significant differences between 240 and 160 as the lower inflorescence dry matter was at least partly compensated by a higher CBD concentration (**Figure 5**) resulting in 95% of the CBD yield using one-third less fertilizer. It is unclear whether this effect is due to enhanced cannabinoid accumulation due to nutrient stress or simply a dilution effect, indicating a maximum production capacity of cannabinoids. Nonetheless, plants experiencing nutrient stress were able to use nutrients more efficiently to produce inflorescence biomass (AE*_inflorescences_*) and CBD yield (AE*_CBDyield_*), mainly due to the higher NUtE for N and K, i.e. already acquired nutrients were re-mobilized from other plant organs. This effect was also reported for other plant species (Kant et al., 2011)

A high concentration of N in the plant does not necessarily correlate with stimulation of the secondary metabolism in cannabis (Saloner and Bernstein, 2021). Rather, the authors suggest a specific impact of N in inflorescences creating a negative correlation between inflorescence N concentration and the production of secondary metabolites not containing N, such as cannabinoids and terpenoids. This correlation is also described as the carbon–nutrient balance, which states that under low N concentration, the production of N-rich primary metabolites and hence growth is restricted, and plant metabolism and energy expenditure shift from creating N-containing metabolites to the production of metabolites that do not contain N, as terpenoids and cannabinoids (Lerdau and Coley, 2002; Song et al., 2023).

The values found for AE in inflorescences are in accordance with the literature (presented as NUE values) ranging from 5 to 17 for the range of fertilizer concentrations between 30 and 320 mg N L^−1^ and a linear decrease of NUE with increasing N concentration was found for liquid mineral fertilizer (Saloner and Bernstein, 2021).

Under mineral fertilization, an optimal N supply range of 160–230 mg N L^−1^ responded best for maximizing inflorescence yield (Bevan et al., 2021), while P was recommended at a rate of 30 mg P L^−1^ (Shiponi and Bernstein, 2021a; Shiponi and Bernstein, 2021b). and 60 mg P L^−1^ (Bevan et al., 2021). For K, the range between 60–175 mg K L^−1^ did not affect plant development but increased K in the leachate indicating a limited K uptake at higher concentrations (Saloner et al., 2019). Nonetheless, it is difficult to compare results to our study – e.g. for optimum fertilization under 160 mg N L^−1^ – due to differences in fertilization regimes, as the experiment by Saloner and Bernstein (2021) was performed with continuous mineral fertigation with drainage, whereas the results by Bevan et al. (2021) are based on a soilless system with replacement of nutrient solution weekly. Thus, it is challenging to calculate exact nutrient inputs and nutrient use efficiencies. In another way, our study highlights the controlled application of fertilizer and induction of nutrient stress only during flowering as a more sustainable fertilization technique to avoid nutrient disposal.

Regarding rates of liquid organic fertilizer, Caplan et al., (2017a; 2017b) recommend a fertilizer rate of 389 mg N L^−1^ (4.0N-1.3P-1.7K) for the vegetative growth stage and 212–261 mg N L^−1^ (2.0N-0.8P-3.3K) for the flowering stage. A positive correlation between fertilizer rate and inflorescence yield but a negative correlation to THCA concentration and yield was found during flowering. This indicates a dilution of compounds with yield increase and points to a reduction of N and P supply during flowering to promote N and P translocation, which can increase utilization and overall agronomic use efficiency.

In our study, the applied nutrient concentrations triggered a nutrient deficiency response, which resulted in an increased production of secondary metabolites as shown by significantly higher CBD concentrations (**Figure 3**) and higher CBD yield agronomic efficiencies (AE*_CBDyield_*). Nutrient deficiency stimulated the translocation of N from older (source) to younger leaves (**Supplementary Table 4**) and generative organs (sinks) (White, 2012). Higher NUtE can be explained by differences in mobilization and translocation of nutrients, which became larger and started earlier with decreasing nutrient concentration, in particular, for N (**Figures 7A**, **8**). These differences in nutrient content over time were to a large degree mediated by the increase in dry matter, but in addition, by higher concentrations in plant organs especially for N and K (**Figures 7A, B, E, F**). At 69 DAP, nutrient contents in inflorescences were not significantly different between treatments with nutrient deprivation stress (**Figure 7**), indicating that nutrient availability was not a limiting factor for the utilization of nutrients during the initial growth of inflorescences. Nevertheless, higher nutrient availability in treatment 240 enhanced biomass production.

Since the inflorescences are commonly the only harvested material in medicinal cannabis cultivation, nutrients accumulated in other plant organs at harvest and in the substrate are unused and discarded. In our study, no significant differences between fertilizer concentrations were found for P concentration in inflorescences at the final harvest, whereas, P concentration in leaves, stem and substrate were significantly higher for treatment 240 (**Supplementary Table 4**). This indicates an overfertilization with P (240 in comparison to 160) at the flowering stage as P utilization by the plants was not increased.

In the experiment from Westmoreland and Bugbee (2022), there was no significant effect of P concentration in inflorescence yield or cannabinoid concentration, but significant differences in leachate P increasing 12-fold in response to the 3-fold increase in P fertilizer rate (25 to 75 mg P L^−1^). The authors suggested an optimum P supply of 25 mg P L^−1^ for continuous mineral fertilization. These values are in line with other studies for CBD- (11 mg P L^−1^) and THC-cultivars (30 mg P L^−1^) (Cockson et al., 2019; Shiponi and Bernstein, 2021b). These studies and our results indicate that maintaining high nutrient levels in leaves and substrate is not relevant at the harvest point. Furthermore, promoting nutrient stress during flowering to enhance P re-mobilization and translocation seems to be a suitable strategy to increase P use efficiency and avoid excessive P fertilization. This is currently a relevant issue to decrease the environmental impact of medicinal cannabis cultivation (Westmoreland and Bugbee, 2022).

### 4.3 Future outlook

The controlled application of fertilizers is paramount to enhancing nutrient efficiency in medicinal cannabis. Differences in cultivars and fertilization regimes make it challenging to compare study results. Experiments are made with different cultivation systems, e.g. continuous fertigation with leaching (Saloner et al., 2019; Saloner and Bernstein, 2021; Westmoreland and Bugbee, 2022) or the controlled fertilizer application in our study. Fertilizer demand can also be genotype-specific and different ranges of optimum nutrient concentrations have been reported for THC- (Bernstein et al., 2019; Shiponi and Bernstein, 2021b) and CBD-rich (Westmoreland and Bugbee, 2022) genotypes. In addition, few studies report the exact amount of nutrients applied and nutrient use efficiency indices. It is important to note that this study is based on a single CBD-rich cannabis chemotype III genotype and for future research, the effect of controlled nutrient stress should be tested within different chemotypes and strains to observe genotype-specific stress responses and nutrient use efficiencies.

The timing of nutrient application and starvation is another important aspect as different fertilizer types have distinct nutrient forms and thus availability over time differs, which has a direct impact on nutrient uptake and use efficiency. Our results indicate, that organic fertilizer should be applied earlier at a higher rate to be available during the final two weeks of flowering when the sink demand for inflorescences is very high. Besides the timing of application, soil amendments, such as plant growth-promoting microorganisms (PGPMs) (i.e., N_2_-fixing and phosphate solubilizing bacteria) and arbuscular mycorrhiza (AM) can also increase nutrient availability of organic fertilizers (Ahmed and Hijri, 2021). Furthermore, the potential of utilizing PGPMs and AMs are manifold, e.g. increase in yield, quality and pathogen resistance as a result of nutrient mobilization, hormone production, disease control and improved stress tolerance (Conant et al., 2017; Backer et al., 2019; Lyu et al., 2019). Nonetheless, it is worth indicating that the application of soil amendments and microorganisms for medicinal cannabis is still under investigation, as results are often contradictory and microbial diversity and efficacy, as microbial communities can be genotype-specific (Winston et al., 2014). Future research should explore if the earlier application of organic fertilizer is sufficient to produce comparable results to mineral fertilizers and whether the application of soil amendments and microorganisms can enhance the bio-availability of nutrients, especially by increasing P uptake efficiency and the conversion of NH_4_^+^ to NO_3_^−^.

The combination of both mineral and organic fertilizers for integrated crop nutrition as performed in our study and also cited in the literature (Bernstein et al., 2019; Da Cunha Leme Filho et al., 2020; Laleh et al., 2021) suggests that the higher availability of mineral fertilizer can be important for plant initial growth, while organic fertilizers can be employed as a more sustainable nutrient complementation during flowering without major yield losses. It is relevant to further investigate the effects of nutrient deprivation in the different stages of flowering, as the major balance between biomass accumulation and the concentration of secondary metabolites can be modulated. Results show that promoting the use of already acquired nutrients in plant increases agronomic use efficiency, but it is still unclear if nutrient deprivation can actually enhance cannabinoid production. Future research should explore the molecular role of nutrient stress in terpenoids and cannabinoids production on trichomes.

Finally, the development of tools for visual assessment of nutrient status in cannabis (e.g., multispectral, hyperspectral) would certainly enable a more flexible adjustment of nutrient inputs according to the actual demand. This would help both producers and as well researchers by facilitating non-destructive analysis with a high temporal resolution.

## 5 Conclusion

Our study showed the potential to reduce fertilizer input while maintaining CBD yield of medicinal cannabis. The decrease in inflorescence yield at the final harvest was compensated by a higher CBD concentration, resulting in 95% of CBD yield using one-third less fertilizer. The utilization efficiency at lower fertilizer rates was increased by a larger re-mobilization and translocation of acquired nutrients. Nutrient acquisition was lower for the organic fertilizer during the final two weeks before harvest, resulting in reduced biomass and CBD yield compared to mineral fertilizer treatments. The fertilizer rate of P can be in general reduced during end of flowering to avoid unproductive nutrient accumulation in vegetative plant organs.

## Data availability statement

The original contributions presented in the study are included in the article/**Supplementary Material**. Further inquiries can be directed to the corresponding author.

## Author contributions

Conceptualization, DM and SG-H; methodology, DM, SM and JH; software, DM, JH; validation, DM, JH, SM and PN; formal analysis, DM; investigation, DM; resources, DM; data curation, DM; writing—original draft preparation, DM; writing—review and editing, JH, SM, PN, and SG-H, visualization, DM; supervision, SM and SG-H; project administration, SG-H; funding acquisition, SG-H. All authors have read and agreed to the published version of the manuscript.

## References

[B1] Aguirre-Becerra H. Vazquez-Hernandez M. C. Saenz de la O D. Alvarado-Mariana A. Guevara-Gonzalez R. G. Garcia-Trejo J. F.. (2021). “Role of stress and defense in plant secondary metabolites production,” in Bioactive Natural Products for Pharmaceutical Applications Advanced Structured Materials. Eds. Pal D. Nayak A. K. (Cham: Springer International Publishing), 151–195. doi: 10.1007/978-3-030-54027-2_5

[B2] Ahmed B. Hijri M. (2021). Potential impacts of soil microbiota manipulation on secondary metabolites production in cannabis. J. Cannabis Res. 3, 25. doi: 10.1186/s42238-021-00082-0 34217364 PMC8254954

[B3] Backer R. Schwinghamer T. Rosenbaum P. McCarty V. Eichhorn Bilodeau S. Lyu D.. (2019). Closing the yield gap for cannabis: a meta-analysis of factors determining cannabis yield. Front. Plant Sci. 0. doi: 10.3389/fpls.2019.00495 PMC6491815 31068957

[B4] Bergstrand K.-J. Löfkvist K. Asp H. (2019). Dynamics of nitrogen availability in pot grown crops with organic fertilization. Biol. Agric. Horticult. 35, 143–150. doi: 10.1080/01448765.2018.1498389

[B5] Bernstein N. Gorelick J. Zerahia R. Koch S. (2019). Impact of N, P, K, and humic acid supplementation on the chemical profile of medical cannabis (Cannabis sativa L). Front. Plant Sci. 10. doi: 10.3389/fpls.2019.00736 PMC6589925 31263470

[B6] Bevan L. Jones M. Zheng Y. (2021)Optimisation of nitrogen, phosphorus, and potassium for soilless production of cannabis sativa in the flowering stage using response surface analysis (Accessed November 17, 2022). 10.3389/fpls.2021.764103 PMC8635921 34868163

[B7] Bilalis D. Krokida M. Roussis I. Papastylianou P. Travlos I. Cheimona N.. (2018). Effects of organic and inorganic fertilization on yield and quality of processing tomato ( *Lycopersicon esculentum* Mill.). Folia Hortic. 30, 321–332. doi: 10.2478/fhort-2018-0027

[B8] Brownlie W. J. Sutton M. A. Reay D. S. Heal K. V. Hermann L. Kabbe C.. (2021). Global actions for a sustainable phosphorus future. Nat. Food 2, 71–74. doi: 10.1038/s43016-021-00232-w 37117414

[B9] Bruce D. Connelly G. Ellison S. (2022). Different Fertility Approaches in Organic Hemp (Cannabis sativa L.) Production Alter Floral Biomass Yield but Not CBD:THC Ratio. Sustainability 14, 6222. doi: 10.3390/su14106222

[B10] Burgel L. Hartung J. Graeff-Hönninger S. (2020). Impact of different growing substrates on growth, yield and cannabinoid content of two cannabis sativa L. Genotypes in a pot culture. Horticulturae 6, 1–14. doi: 10.3390/horticulturae6040062 PMC7355821 32521804

[B11] Caplan D. Dixon M. Zheng Y. (2017a). Optimal rate of organic fertilizer during the flowering stage for cannabis grown in two coir-based substrates. horts 52, 1796–1803. doi: 10.21273/HORTSCI12401-17

[B12] Caplan D. Dixon M. Zheng Y. (2017b). Optimal rate of organic fertilizer during the vegetative-stage for cannabis grown in two coir-based substrates. horts 52, 1307–1312. doi: 10.21273/HORTSCI11903-17

[B13] CBD. (2022). Report of the Open-ended Working Group on the Post-2020 Global Biodiversity Framework on its third meeting (Part II). Geneva, Switzerland: Convention on Biological Diversity - UN Environmental Programme. Available at: https://www.cbd.int/doc/c/50c9/a685/3844e4030802e9325bc5e0b4/wg2020-03-07-en.pdf.

[B14] Cervantes J. (2006). Marijuana horticulture the indoor/outdoor medical grower’s bible (Portland: Van Patten Publishing,U.S).

[B15] Chang E.-H. Chung R.-S. Tsai Y.-H. (2007). Effect of different application rates of organic fertilizer on soil enzyme activity and microbial population. Soil Sci. Plant Nutr. 53, 132–140. doi: 10.1111/j.1747-0765.2007.00122.x

[B16] Cockson P. Landis H. Smith T. Hicks K. Whipker B. E. (2019). Characterization of nutrient disorders of Cannabis sativa. Appl. Sci. 9, 4432. doi: 10.3390/app9204432

[B17] Conant R. T. Walsh R. P. Walsh M. Bell C. W. Wallenstein M. D. (2017). Effects of a microbial biostimulant, mammoth PTM, on Cannabis sativa bud yield. J. Hortic. 4. doi: 10.4172/2376-0354.1000191

[B18] Congreves K. A. Otchere O. Ferland D. Farzadfar S. Williams S. Arcand M. M. (2021)Nitrogen use efficiency definitions of today and tomorrow (Accessed March 31, 2023). 10.3389/fpls.2021.637108 PMC8220819 34177975

[B19] Crispim Massuela D. Hartung J. Munz S. Erpenbach F. Graeff-Hönninger S. (2022). Impact of harvest time and pruning technique on total CBD concentration and yield of medicinal cannabis. Plants 11, 140. doi: 10.3390/plants11010140 35009146 PMC8747189

[B20] Da Cunha Leme Filho J. F. Thomason W. E. Evanylo G. K. Zhang X. Strickland M. S. Chim B. K.. (2020). Biochemical and physiological responses of Cannabis sativa to an integrated plant nutrition system. Agron. J. 112, 5237–5248. doi: 10.1002/agj2.20400

[B21] Decorte T. Potter G. R. (2015). The globalisation of cannabis cultivation: A growing challenge. Int. J. Drug Policy 26, 221–225. doi: 10.1016/j.drugpo.2014.12.011 25638581

[B22] Drotleff L. (2022) Sustainable cannabis fertilizer options as prices rise for key nutrition providers (MJBizDaily) (Accessed November 10, 2022).

[B23] Eisa M. Ragauskaitė D. Adhikari S. Bella F. Baltrusaitis J. (2022). Role and responsibility of sustainable chemistry and engineering in providing safe and sufficient nitrogen fertilizer supply at turbulent times. ACS Sustain. Chem. Eng. 10, 8997–9001. doi: 10.1021/acssuschemeng.2c03972

[B24] Hirose T. (2011). Nitrogen use efficiency revisited. Oecologia 166, 863–867. doi: 10.1007/s00442-011-1942-z 21359566

[B25] Ii S. L. A. Pearson B. Kjelgren R. Brym Z. (2021). Response of essential oil hemp (Cannabis sativa L.) growth, biomass, and cannabinoid profiles to varying fertigation rates. PloS One 16, e0252985. doi: 10.1371/journal.pone.0252985 34324496 PMC8320997

[B26] Jacoby R. Peukert M. Succurro A. Koprivova A. Kopriva S. (2017). The role of soil microorganisms in plant mineral nutrition—Current knowledge and future directions. Front. Plant Sci. 8. doi: 10.3389/fpls.2017.01617 PMC5610682 28974956

[B27] Jin D. Jin S. Chen J. (2019). Cannabis indoor growing conditions, management practices, and post-harvest treatment: a review. Am. J. Plant Sci. 10, 925. doi: 10.4236/ajps.2019.106067

[B28] Kalinowski J. Edmisten K. Davis J. McGinnis M. Hicks K. Cockson P.. (2020). Augmenting nutrient acquisition ranges of greenhouse grown CBD (Cannabidiol) hemp (Cannabis sativa) cultivars. Horticulturae 6, 98. doi: 10.3390/horticulturae6040098

[B29] Kant S. Bi Y.-M. Rothstein S. J. (2011). Understanding plant response to nitrogen limitation for the improvement of crop nitrogen use efficiency. J. Exp. Bot. 62, 1499–1509. doi: 10.1093/jxb/erq297 20926552

[B30] Laleh S. Jami Al-Ahmadi M. Parsa S. (2021). Response of hemp (Cannabis sativa L.) to integrated application of chemical and manure fertilizers. Acta Agric. Slov. 117, 1. doi: 10.14720/aas.2021.117.2.1819

[B31] Landi S. Berni R. Capasso G. Hausman J.-F. Guerriero G. Esposito S. (2019). Impact of nitrogen nutrition on Cannabis sativa: an update on the current knowledge and future prospects. Int. J. Mol. Sci. 20, 5803. doi: 10.3390/ijms20225803 31752217 PMC6888403

[B32] Lerdau M. Coley P. D. (2002). Benefits of the carbon-nutrient balance hypothesis. Oikos 98, 534–536. doi: 10.1034/j.1600-0706.2002.980318.x

[B33] Lowenfels J. (2013). Teaming with Nutrients: The Organic Gardener’s Guide to Optimizing Plant Nutrition. Illustrated edition (Portland, Or: Timber Press).

[B34] Lowenfels J. Lewis W. (2010). Teaming with Microbes: The Organic Gardener’s Guide to the Soil Food Web, Revised Edition. (Portland, Or: Timber Press).

[B35] Lyu D. Backer R. Robinson W. G. Smith D. L. (2019). Plant growth-promoting rhizobacteria for cannabis production: yield, cannabinoid profile and disease resistance. Front. Microbiol. 10. doi: 10.3389/fmicb.2019.01761 PMC6698789 31456755

[B36] Madhusoodanan J. (2019). Can cannabis go green? Nature 572, S8–S9. doi: 10.1038/d41586-019-02526-3 31462788

[B37] Malík M. Velechovský J. Tlustoš P. (2021). The overview of existing knowledge on medical cannabis plants growing. Plant Soil Environ. 67 (2021), 425–442. doi: 10.17221/96/2021-PSE

[B38] Maschner H. (1986) Mineral Nutrition in Higher Plants (Stuttgart: Academic Press) (Accessed December 9, 2022).

[B39] Mayer B. F. Ali-Benali M. A. Demone J. Bertrand A. Charron J.-B. (2015). Cold acclimation induces distinctive changes in the chromatin state and transcript levels of COR genes in Cannabis sativa varieties with contrasting cold acclimation capacities. Physiol. Plant. 155, 281–295. doi: 10.1111/ppl.12318 25534661

[B40] Menz J. Range T. Trini J. Ludewig U. Neuhäuser B. (2018). Molecular basis of differential nitrogen use efficiencies and nitrogen source preferences in contrasting Arabidopsis accessions. Sci. Rep. 8, 3373. doi: 10.1038/s41598-018-21684-4 29463824 PMC5820274

[B41] Mills E. (2012). The carbon footprint of indoor Cannabis production. Energy Policy 46, 58–67. doi: 10.1016/j.enpol.2012.03.023

[B42] Nemati R. Fortin J.-P. Craig J. Donald S. (2021). Growing mediums for medical cannabis production in North America. Agronomy 11, 1366. doi: 10.3390/agronomy11071366

[B43] Pagnani G. Pellegrini M. Galieni A. D’Egidio S. Matteucci F. Ricci A.. (2018). Plant growth-promoting rhizobacteria (PGPR) in Cannabis sativa ‘Finola’ cultivation: An alternative fertilization strategy to improve plant growth and quality characteristics. Ind. Crops Prod. 123, 75–83. doi: 10.1016/j.indcrop.2018.06.033

[B44] Piepho H. P. Büchse A. Emrich K. (2003). A hitchhiker’s guide to mixed models for randomized experiments. J. Agron. Crop Sci. 189, 310–322. doi: 10.1046/j.1439-037X.2003.00049.x

[B45] Reichel P. Munz S. Hartung J. KotIranta S. Graeff-Hönninger S. (2022). Impacts of different light spectra on CBD, CBDA and terpene concentrations in relation to the flower positions of different Cannabis sativa L. Strains. Plants 11, 2695. doi: 10.3390/plants11202695 36297719 PMC9612076

[B46] Saloner A. Bernstein N. (2021). Nitrogen supply affects cannabinoid and terpenoid profile in medical cannabis (Cannabis sativa L.). Ind. Crops Prod. 167, 113516. doi: 10.1016/j.indcrop.2021.113516

[B47] Saloner A. Bernstein N. (2022). Nitrogen Source Matters: High NH4/NO3 Ratio Reduces Cannabinoids, Terpenoids, and Yield in Medical Cannabis. Front. Plant Sci. 13. doi: 10.3389/fpls.2022.830224 PMC9198551 35720524

[B48] Saloner A. Sacks M. M. Bernstein N. (2019). Response of medical cannabis (Cannabis sativa L.) genotypes to K supply under long photoperiod. Front. Plant Sci. 10. doi: 10.3389/fpls.2019.01369 PMC6876614 31803198

[B49] Schachtman D. P. Reid R. J. Ayling S. M. (1998). Phosphorus uptake by plants: from soil to cell. Plant Physiol. 116, 447–453. doi: 10.1104/pp.116.2.447 9490752 PMC1539172

[B50] Shiponi S. Bernstein N. (2021a). Response of medical cannabis (Cannabis sativa L.) genotypes to P supply under long photoperiod: Functional phenotyping and the ionome. Ind. Crops Prod. 161, 113154. doi: 10.1016/j.indcrop.2020.113154

[B51] Shiponi S. Bernstein N. (2021b). The highs and lows of P supply in medical cannabis: effects on cannabinoids, the ionome, and morpho-physiology. Front. Plant Sci. 12. doi: 10.3389/fpls.2021.657323 PMC8320666 34335641

[B52] Song C. Saloner A. Fait A. Bernstein N. (2023). Nitrogen deficiency stimulates cannabinoid biosynthesis in medical cannabis plants by inducing a metabolic shift towards production of low-N metabolites. Ind. Crops Prod. 202, 116969. doi: 10.1016/j.indcrop.2023.116969

[B53] Wartenberg A. C. Holden P. A. Bodwitch H. Parker-Shames P. Novotny T. Harmon T. C.. (2021). Cannabis and the environment: what science tells us and what we still need to know. Environ. Sci. Technol. Lett. 8, 98–107. doi: 10.1021/acs.estlett.0c00844

[B54] Westmoreland F. M. Bugbee B. (2022). Sustainable Cannabis Nutrition: Elevated root-zone phosphorus significantly increases leachate P and does not improve yield or quality. Front. Plant Sci. 13. doi: 10.3389/fpls.2022.1015652 PMC9724152 36483962

[B55] White P. J. (2012). “Chapter 3 - long-distance transport in the xylem and phloem,” in Marschner’s Mineral Nutrition of Higher Plants, 3rd ed. Ed. Marschner P. (San Diego: Academic Press), 49–70. doi: 10.1016/B978-0-12-384905-2.00003-0

[B56] Wink M. (2008). Plant secondary metabolism: diversity, function and its evolution. Natural Prod. Commun. 3, 1934578X0800300801. doi: 10.1177/1934578X0800300801

[B57] Winston M. E. Hampton-Marcell J. Zarraonaindia I. Owens S. M. Moreau C. S. Gilbert J. A.. (2014). Understanding cultivar-specificity and soil determinants of the cannabis microbiome. PloS One 9, e99641. doi: 10.1371/journal.pone.0099641 24932479 PMC4059704

[B58] Ye J. Y. Tian W. H. Jin C. W. (2022). Nitrogen in plants: from nutrition to the modulation of abiotic stress adaptation. Stress Biol. 2, 4. doi: 10.1007/s44154-021-00030-1 37676383 PMC10441927

[B59] Zheng Z. Fiddes K. Yang L. (2021). A narrative review on environmental impacts of cannabis cultivation. J. Cannabis Res. 3, 35. doi: 10.1186/s42238-021-00090-0 34362475 PMC8349047

[B60] Zhu X. Ros G. H. Xu M. Cai Z. Sun N. Duan Y.. (2023). Long-term impacts of mineral and organic fertilizer inputs on nitrogen use efficiency for different cropping systems and site conditions in Southern China. Eur. J. Agron. 146, 126797. doi: 10.1016/j.eja.2023.126797

