# Supplementary material for: Cannabis Hunger Games: nutrient stress induction in flowering stage – impact of organic and mineral fertilizer levels on biomass, cannabidiol (CBD) yield and nutrient use efficiency
Source: Front Plant Sci. 2023 Sep 19;14:1233232. doi: 10.3389/fpls.2023.1233232 (PMC10547009; doi:10.3389/fpls.2023.1233232)
Supplement: Supplementary file 1 [file DataSheet_1.docx]

Supplementary Material

# Supplementary Data

Not applicable.

# Supplementary Figures and Tables

Supplementary Table 1. Daily values of average, minimum and maximum air temperature (°C) and relative humidity (%) for the growing room during the cultivation period.

|  | **Room temperature (°C)** | | | **Air humidity (%)** | | |
| --- | --- | --- | --- | --- | --- | --- |
| **Date** | **Minimum** | **Average** | **Maximum** | **Minimum** | **Average** | **Maximum** |
| 07/10/2021 | 20.5 | 23.7 | 25.6 | 36.8 | 49.6 | 67.3 |
| 08/10/2021 | 20.9 | 23.3 | 24.3 | 40.6 | 52.2 | 65.2 |
| 09/10/2021 | 19.2 | 22.8 | 25.7 | 37.1 | 52.4 | 67.5 |
| 10/10/2021 | 19 | 22.8 | 25.7 | 36.5 | 51.8 | 67.4 |
| 11/10/2021 | 19.4 | 23.2 | 25.8 | 33.8 | 52.3 | 67.7 |
| 12/10/2021 | 20.2 | 23.4 | 25.9 | 36.6 | 54.3 | 68.3 |
| 13/10/2021 | 19.4 | 22.7 | 25.3 | 37.2 | 52.0 | 67.4 |
| 14/10/2021 | 19.3 | 23.0 | 25.9 | 31.2 | 50.0 | 67.4 |
| 15/10/2021 | 19.4 | 23.0 | 25.7 | 36.8 | 53.2 | 67.5 |
| 16/10/2021 | 20 | 23.1 | 25.8 | 37 | 54.1 | 67.4 |
| 17/10/2021 | 19.6 | 23.0 | 25.6 | 38.8 | 54.6 | 67.7 |
| 18/10/2021 | 20 | 23.0 | 25.3 | 36.5 | 54.2 | 67.4 |
| 19/10/2021 | 20 | 23.3 | 26.1 | 47.5 | 57.5 | 67.7 |
| 20/10/2021 | 21.2 | 23.7 | 25.1 | 40.8 | 54.4 | 68.1 |
| 21/10/2021 | 19.7 | 23.7 | 29.1 | 34.5 | 49.2 | 67.5 |
| 22/10/2021 | 20.2 | 23.5 | 25.9 | 26 | 45.7 | 67.4 |
| 23/10/2021 | 19.3 | 23.4 | 26.4 | 33.5 | 46.5 | 67.4 |
| 24/10/2021 | 18.5 | 23.6 | 26.5 | 34.3 | 48.8 | 67 |
| 25/10/2021 | 19.8 | 24.3 | 28.7 | 32.3 | 48.7 | 67.5 |
| 26/10/2021 | 20.2 | 24.0 | 27.2 | 38.5 | 50.0 | 67.2 |
| 27/10/2021 | 20.2 | 23.8 | 26.5 | 37 | 52.2 | 68.8 |
| 28/10/2021 | 21 | 24.0 | 26.3 | 38.8 | 53.7 | 70.2 |
| 29/10/2021 | 20.7 | 24.0 | 26 | 36.5 | 51.1 | 68.6 |
| 30/10/2021 | 20.4 | 24.2 | 27.5 | 39.8 | 52.2 | 67.9 |
| 31/10/2021 | 21.3 | 24.4 | 26.8 | 38 | 51.3 | 67.5 |
| 01/11/2021 | 20.8 | 24.2 | 26.6 | 42.6 | 54.5 | 68.2 |
| 02/11/2021 | 20.9 | 23.9 | 27.2 | 44.2 | 55.0 | 68.8 |
| 03/11/2021 | 20.7 | 23.9 | 26.5 | 42.3 | 53.5 | 69.9 |
| 04/11/2021 | 21 | 23.3 | 24.8 | 38 | 52.5 | 68.7 |
| 05/11/2021 | 20.5 | 23.8 | 26.1 | 38 | 50.9 | 68.6 |
| 06/11/2021 | 20.6 | 23.7 | 25.1 | 37.7 | 51.2 | 69.6 |
| 07/11/2021 | 19.8 | 24.2 | 29.3 | 36.4 | 48.9 | 68.1 |
| 08/11/2021 | 19.9 | 23.7 | 26.2 | 31.6 | 48.7 | 67.5 |
| 09/11/2021 | 20.8 | 24.2 | 28.8 | 36.6 | 50.7 | 69.1 |
| 10/11/2021 | 19.2 | 24.0 | 29.4 | 36.9 | 50.5 | 68 |
| 11/11/2021 | 19.5 | 23.5 | 28.5 | 36.4 | 53.3 | 67.9 |
| 12/11/2021 | 19.1 | 22.9 | 29.1 | 36.8 | 56.6 | 68.2 |
| 13/11/2021 | 19.9 | 23.1 | 26.8 | 39.4 | 57.6 | 69.1 |
| 14/11/2021 | 19.8 | 22.4 | 24.4 | 39 | 56.9 | 68.1 |
| 15/11/2021 | 18.5 | 21.5 | 24 | 38.1 | 56.9 | 66.2 |
| 16/11/2021 | 19.7 | 22.7 | 24.8 | 44.2 | 59.5 | 68.2 |
| 17/11/2021 | 20.9 | 22.9 | 25.9 | 47.2 | 61.7 | 75.7 |
| 18/11/2021 | 21 | 23.1 | 25.1 | 45.3 | 61.0 | 70.2 |
| 19/11/2021 | 21 | 23.1 | 28.1 | 42.5 | 61.6 | 69.7 |
| 20/11/2021 | 20.8 | 22.8 | 24.5 | 47.4 | 61.5 | 69.4 |
| 21/11/2021 | 20.7 | 22.6 | 24.1 | 43.6 | 61.0 | 70.8 |
| 22/11/2021 | 18.3 | 21.8 | 24.4 | 45.7 | 61.0 | 69.1 |
| 23/11/2021 | 18.1 | 21.5 | 26.5 | 40.4 | 61.0 | 68.4 |
| 24/11/2021 | 18.9 | 21.8 | 26.9 | 46.9 | 62.1 | 69.5 |
| 25/11/2021 | 20.7 | 23.1 | 27.6 | 43.1 | 62.0 | 71 |
| 26/11/2021 | 19.4 | 22.9 | 26.7 | 49.4 | 63.4 | 71.8 |
| 27/11/2021 | 18.4 | 22.1 | 24.4 | 52.1 | 62.3 | 70.8 |
| 28/11/2021 | 21.3 | 23.2 | 27.5 | 49.2 | 63.6 | 71.5 |
| 29/11/2021 | 20.3 | 22.6 | 27.8 | 40.9 | 61.0 | 71.8 |
| 30/11/2021 | 20 | 22.3 | 26.8 | 48 | 63.1 | 70.8 |
| 01/12/2021 | 18.4 | 21.4 | 26.4 | 44.4 | 59.8 | 70.9 |
| 02/12/2021 | 20.4 | 22.1 | 24.1 | 49.3 | 63.6 | 71.9 |
| 03/12/2021 | 20.3 | 22.7 | 27.1 | 42.1 | 62.0 | 71.2 |
| 04/12/2021 | 18.5 | 21.8 | 23.9 | 48.7 | 63.0 | 73.6 |
| 05/12/2021 | 20.1 | 22.7 | 26.7 | 45.1 | 61.8 | 71.9 |
| 06/12/2021 | 21.2 | 22.8 | 25.2 | 43.3 | 61.7 | 70.9 |
| 07/12/2021 | 21 | 22.6 | 25.5 | 39.7 | 62.6 | 72.9 |
| 08/12/2021 | 19.2 | 22.8 | 25.5 | 49.4 | 62.4 | 73.1 |
| 09/12/2021 | 18.4 | 22.1 | 24.2 | 40.4 | 60.1 | 70.5 |
| 10/12/2021 | 18.4 | 21.8 | 28.3 | 43.8 | 60.5 | 68.6 |
| 11/12/2021 | 18.2 | 21.0 | 24 | 43.4 | 60.6 | 69.8 |
| 12/12/2021 | 18.5 | 22.2 | 27.4 | 39.5 | 59.2 | 70 |
| 13/12/2021 | 18.5 | 22.2 | 24.7 | 44.7 | 60.0 | 69.3 |
| 14/12/2021 | 19.7 | 22.1 | 24.6 | 41.3 | 59.4 | 67.9 |
| 15/12/2021 | 18.8 | 21.9 | 24.5 | 36.9 | 57.4 | 69.8 |
| 16/12/2021 | 19 | 22.0 | 24.2 | 41.9 | 59.8 | 72 |
| 17/12/2021 | 19.1 | 21.8 | 23.9 | 40 | 59.4 | 70.6 |
| 18/12/2021 | 18.4 | 21.2 | 23.9 | 37.6 | 57.9 | 67.4 |
| 19/12/2021 | 18.5 | 21.6 | 25.8 | 38.3 | 59.6 | 69.5 |
| 20/12/2021 | 19.1 | 21.9 | 25 | 43.5 | 59.4 | 68.7 |
| 21/12/2021 | 18 | 21.6 | 27.7 | 39.8 | 59.4 | 67.2 |
| 22/12/2021 | 17.9 | 22.2 | 29.3 | 48.1 | 60.6 | 68.8 |
| 23/12/2021 | 18.2 | 21.8 | 29.3 | 36.7 | 57.7 | 67.1 |
| 24/12/2021 | 18.4 | 21.6 | 25.6 | 44.4 | 60.1 | 68.1 |
| 25/12/2021 | 20.1 | 22.7 | 26.4 | 41.6 | 60.2 | 68.1 |
| 26/12/2021 | 19.9 | 22.5 | 25.8 | 43.3 | 59.6 | 67.8 |
| 27/12/2021 | 19.1 | 22.5 | 26.2 | 40.1 | 58.5 | 67.9 |
| 28/12/2021 | 18.3 | 21.5 | 25.3 | 36.9 | 57.7 | 67.4 |
| 29/12/2021 | 18.5 | 22.0 | 26.2 | 41.3 | 57.2 | 68.4 |
| 30/12/2021 | 19.5 | 22.9 | 26.1 | 45.9 | 59.4 | 68 |
| 31/12/2021 | 19.2 | 22.5 | 25.9 | 37.3 | 57.8 | 68.3 |
| 01/01/2022 | 18.5 | 21.9 | 25.3 | 37.2 | 57.9 | 67.4 |
| 02/01/2022 | 18.9 | 22.0 | 25.1 | 36.9 | 56.3 | 68.1 |
| 03/01/2022 | 18.4 | 21.4 | 24.5 | 37.4 | 54.1 | 65.8 |
| 04/01/2022 | 18.3 | 21.2 | 24.9 | 36.3 | 57.8 | 65.8 |
| 05/01/2022 | 18.3 | 20.7 | 23.4 | 36.6 | 56.7 | 66 |
| 06/01/2022 | 18.2 | 21.4 | 25.7 | 37.3 | 58.0 | 68.6 |
| 07/01/2022 | 18.2 | 21.7 | 27.8 | 36.7 | 57.8 | 68.2 |
| 08/01/2022 | 18.3 | 21.3 | 25.9 | 36.7 | 57.0 | 67.6 |
| 09/01/2022 | 18.2 | 21.1 | 25 | 36.6 | 55.9 | 67.8 |
| 10/01/2022 | 19.1 | 22.2 | 24.6 | 36.9 | 57.1 | 69.9 |
| 11/01/2022 | 18.3 | 21.5 | 23.9 | 36.5 | 54.3 | 68 |
| 12/01/2022 | 18.1 | 21.8 | 28.5 | 29 | 55.7 | 66.4 |
| 13/01/2022 | 18.4 | 18.4 | 18.4 | 65.4 | 65.4 | 65.4 |

Supplementary Table 2. Fertilization sequence with amount of fertilizer solution and nutrients provided to each plant at each fertilization event.

| **DAP** | **Cultivation week** | **Fertilizer solution amount (ml plant^-1^)** | **Cumulative solution amount (ml plant^-1^)** | **Fertilizer concentration (mg L^-1^)** | | | | | | | |  |
| --- | --- | --- | --- | --- | --- | --- | --- | --- | --- | --- | --- | --- |
|  |  |  |  | **80** | | | **160** | | | **240** | | |
|  |  |  |  | **Fertilizer amount (mg plant^-1^)** | | | | | | | |  |
|  |  |  |  | **N** | **P** | **K** | **N** | **P** | **K** | **N** | **P** | **K** |
| **7** | **1** | 0 | 0 | 0 | 0 | 0 | 0 | 0 | 0 | 0 | 0 | 0 |
| **15** | **2** | 0 | 0 | 0 | 0 | 0 | 0 | 0 | 0 | 0 | 0 | 0 |
| **22** | **3** | 100 | 100 | 8 | 3.2 | 8 | 16 | 6.4 | 16 | 24 | 9.6 | 24 |
| **27** | **4** | 100 | 200 | 8 | 3.2 | 8 | 16 | 6.4 | 16 | 24 | 9.6 | 24 |
| **29** | **4** | 100 | 300 | 8 | 3.2 | 8 | 16 | 6.4 | 16 | 24 | 9.6 | 24 |
| **33** | **5** | 150 | 450 | 12 | 4.8 | 12 | 24 | 9.6 | 24 | 36 | 14.4 | 36 |
| **36** | **5** | 150 | 600 | 12 | 4.8 | 12 | 24 | 9.6 | 24 | 36 | 14.4 | 36 |
| **41** | **6** | 200 | 800 | 16 | 6.4 | 16 | 32 | 12.8 | 32 | 48 | 19.2 | 48 |
| **44** | **6** | 200 | 1000 | 16 | 6.4 | 16 | 32 | 12.8 | 32 | 48 | 19.2 | 48 |
| **47** | **7** | 300 | 1300 | 24 | 9.6 | 24 | 48 | 19.2 | 48 | 72 | 28.8 | 72 |
| **50** | **7** | 300 | 1600 | 24 | 9.6 | 24 | 48 | 19.2 | 48 | 72 | 28.8 | 72 |
| **55** | **8** | 400 | 2000 | 32 | 12.8 | 32 | 64 | 25.6 | 64 | 96 | 38.4 | 96 |
| **58** | **8** | 400 | 2400 | 32 | 12.8 | 32 | 64 | 25.6 | 64 | 96 | 38.4 | 96 |
| **61** | **9** | 500 | 2900 | 40 | 16 | 40 | 80 | 32 | 80 | 120 | 48 | 120 |
| **64** | **9** | 500 | 3400 | 40 | 16 | 40 | 80 | 32 | 80 | 120 | 48 | 120 |
| **69** | **10** | 600 | 4000 | 48 | 19.2 | 48 | 96 | 38.4 | 96 | 144 | 57.6 | 144 |
| **72** | **10** | 600 | 4600 | 48 | 19.2 | 48 | 96 | 38.4 | 96 | 144 | 57.6 | 144 |
| **75** | **11** | 600 | 5200 | 48 | 19.2 | 48 | 96 | 38.4 | 96 | 144 | 57.6 | 144 |
| **77** | **11** | 600 | 5800 | 48 | 19.2 | 48 | 96 | 38.4 | 96 | 144 | 57.6 | 144 |
| **82** | **12** | 500 | 6300 | 40 | 16 | 40 | 80 | 32 | 80 | 120 | 48 | 120 |
| **85** | **12** | 500 | 6800 | 40 | 16 | 40 | 80 | 32 | 80 | 120 | 48 | 120 |
| **88** | **13** | 400 | 7200 | 32 | 12.8 | 32 | 64 | 25.6 | 64 | 96 | 38.4 | 96 |
| **92** | **13** | 400 | 7600 | 32 | 12.8 | 32 | 64 | 25.6 | 64 | 96 | 38.4 | 96 |
| **99** | **14** | 0 | **total** | **608** | **243.2** | **608** | **1216** | **486.4** | **1216** | **1824** | **729.6** | **1824** |

Supplementary Table 3. Average values ± Standard Errors for pH and EC for each of the fertilizer treatments

| **Fertilizer Type** | **Fertilizer Concentration** | **pH** | **EC** |
| --- | --- | --- | --- |
| **Organic** | **80** | 6.85 ± 1.70 | 0.70 ± 0.02 |
|  | **160** | 7.18 ± 0.40 | 1.26 ± 0.04 |
|  | **240** | 7.16 ± 0.32 | 1.77 ± 0.03 |
| **Mineral** | **80** | 8.74 ± 0.27 | 0.76 ± 0.03 |
|  | **160** | 8.57 ± 0.29 | 1.37 ± 0.02 |
|  | **240** | 8.52 ± 0.29 | 1.92 ± 0.03 |

Supplementary Table 4. Least square means (±standard error) of N, P and K concentration [%] for the fertilizer treatments (80, 160 and 240 mg N L^-1^) for inflorescences, leaves, stem, and the substrate at different harvest dates (Days after planting, DAP). Means with at least one identical letter are not significantly different from each other according to Fisher’s LSD test with α =0.05.

|  | **Nutrient** | **Fert Conc.** | **DAP** | | | | | |
| --- | --- | --- | --- | --- | --- | --- | --- | --- |
|  |  |  | **22** | **36** | **54** | **69** | **83** | **99** |
| **INFLORESCENCES** | **N** | **80** |  |  | 3.72 ± 0.07 b | 3.33 ± 0.06 c | 3.51 ± 0.08 c | 3.43 ± 0.04 c |
|  |  | **160** |  |  | 4.08 ± 0.07 a | 3.84 ± 0.06 b | 3.89 ± 0.08 b | 3.89 ± 0.04 b |
|  |  | **240** |  |  | 4.17 ± 0.07 a | 4.22 ± 0.06 a | 4.41 ± 0.08 a | 4.35 ± 0.04 a |
|  | **P** | **80** |  |  | 0.73 ± 0.02 | 0.79 ± 0.01 b | 0.88 ± 0.01 b | 0.94 ± 0.01 a |
|  |  | **160** |  |  | 0.77 ± 0.02 | 0.84 ± 0.01 a | 0.88 ± 0.01 b | 0.95 ± 0.01 a |
|  |  | **240** |  |  | 0.75 ± 0.02 | 0.86 ± 0.01 a | 0.94 ± 0.01 a | 0.95 ± 0.01 a |
|  | **K** | **80** |  |  | 2.75 ± 0.04 | 2.36 ± 0.02 | 2.39 ± 0.06 | 2.26 ± 0.02 |
|  |  | **160** |  |  | 2.84 ± 0.04 | 2.6 ± 0.02 | 2.47 ± 0.06 | 2.44 ± 0.02 |
|  |  | **240** |  |  | 2.91 ± 0.04 | 2.71 ± 0.02 | 2.74 ± 0.06 | 2.63 ± 0.02 |
| **LEAVES** | **N** | **80** | 4.85 ± 0.21 | 4.42 ± 0.11 ab | 2.8 ± 0.13 b | 1.77 ± 0.06 c | 1.5 ± 0.16 b | 0.96 ± 0.06 c |
|  |  | **160** | 4.86 ± 0.21 | 4.12 ± 0.11 b | 3.23 ± 0.13 ab | 2.32 ± 0.06 b | 2.03 ± 0.16 b | 1.34 ± 0.06 b |
|  |  | **240** | 4.83 ± 0.21 | 4.61 ± 0.11 a | 3.39 ± 0.13 a | 3.12 ± 0.06 a | 3 ± 0.16 a | 2.09 ± 0.06 a |
|  | **P** | **80** | 0.59 ± 0.02 | 0.6 ± 0.02 | 0.51 ± 0.02 | 0.45 ± 0.01 b | 0.49 ± 0.03 b | 0.41 ± 0.01 b |
|  |  | **160** | 0.6 ± 0.02 | 0.54 ± 0.02 | 0.58 ± 0.02 | 0.5 ± 0.01 b | 0.47 ± 0.03 b | 0.43 ± 0.01 b |
|  |  | **240** | 0.59 ± 0.02 | 0.6 ± 0.02 | 0.56 ± 0.02 | 0.59 ± 0.01 a | 0.58 ± 0.03 a | 0.47 ± 0.01 a |
|  | **K** | **80** | 2.68 ± 0.07 | 2.63 ± 0.05 | 2.26 ± 0.05 b | 1.79 ± 0.05 c | 1.66 ± 0.17 b | 1.5 ± 0.09 b |
|  |  | **160** | 2.72 ± 0.07 | 2.58 ± 0.05 | 2.45 ± 0.05 b | 2.09 ± 0.05 b | 1.89 ± 0.17 ab | 1.74 ± 0.09 b |
|  |  | **240** | 2.64 ± 0.07 | 2.64 ± 0.05 | 2.5 ± 0.05 a | 2.58 ± 0.05 a | 2.45 ± 0.17 a | 2.28 ± 0.09 a |
| **STEMS** | **N** | **80** | 3 ± 0.1 |  |  |  |  | 0.7 ± 0.09 |
|  |  | **160** | 3.19 ± 0.1 |  |  |  |  | 0.77 ± 0.09 |
|  |  | **240** | 3.08 ± 0.1 |  |  |  |  | 0.98 ± 0.09 |
|  | **P** | **80** | 0.46 ± 0.01 |  |  |  |  | 0.25 ± 0.02 c |
|  |  | **160** | 0.47 ± 0.01 |  |  |  |  | 0.34 ± 0.02 b |
|  |  | **240** | 0.45 ± 0.01 |  |  |  |  | 0.45 ± 0.02 a |
|  | **K** | **80** | 3.98 ± 0.11 |  |  |  |  | 0.98 ± 0.05 |
|  |  | **160** | 4.11 ± 0.11 |  |  |  |  | 0.97 ± 0.05 |
|  |  | **240** | 4.13 ± 0.11 |  |  |  |  | 1.27 ± 0.05 |
| **SUBSTRATE** | **N** | **80** |  |  |  |  |  | 0.75 ± 0.02 b |
|  |  | **160** |  |  |  |  |  | 0.82 ± 0.02 a |
|  |  | **240** |  |  |  |  |  | 0.85 ± 0.02 a |
|  | **P** | **80** |  |  |  |  |  | 0.15 ± 0 c |
|  |  | **160** |  |  |  |  |  | 0.17 ± 0 b |
|  |  | **240** |  |  |  |  |  | 0.21 ± 0 a |
|  | **K** | **80** |  |  |  |  |  | 0.38 ± 0.03 b |
|  |  | **160** |  |  |  |  |  | 0.47 ± 0.03 a |
|  |  | **240** |  |  |  |  |  | 0.5 ± 0.03 a |

Supplementary Table 5. SPAD values presented for fertilizer type (organic, mineral) and concentration (80, 160, and 240 mg N L^-1^) for leaves at three canopy positions of the plant (top, mid, low) measured at the first (36 Days after planting, DAP) and last harvest (99 DAP) date during flowering. Reduction was calculated for the change from first to last harvest date.

| **Fertilizer Type** | **Fertilizer Concentration** | **Leaf position** | **SPAD value** | | |
| --- | --- | --- | --- | --- | --- |
|  |  |  | **36 DAP** | **99 DAP** | **Reduction (%)** |
| **Organic** | **80** | **Top** | 51.4 ± 1.4 | 10.4 ± 4.8 | 79.8 |
|  |  | **Mid** | 59.4 ± 5.6 | 3.5 ± 2.4 | 94.1 |
|  |  | **Low** | 56.6 ± 3.3 | 7.8 ± 9.5 | 86.2 |
|  | **160** | **Top** | 53.6 ± 1.2 | 31.5 ± 5.9 | 41.3 |
|  |  | **Mid** | 57.8 ± 2.2 | 25.9 ± 7.3 | 55.2 |
|  |  | **Low** | 53.9 ± 4.2 | 21.9 ± 7.2 | 59.3 |
|  | **240** | **Top** | 55.3 ± 4.6 | 42.3 ± 5.5 | 23.6 |
|  |  | **Mid** | 56.8 ± 2.9 | 39.3 ± 6.5 | 30.8 |
|  |  | **Low** | 54.8 ± 3.5 | 32.7 ± 8.7 | 40.3 |
| **Mineral** | **80** | **Top** | 54.6 ± 2.6 | 8.2 ± 3.5 | 84.9 |
|  |  | **Mid** | 55.5 ± 3.0 | 4.9 ± 5.0 | 91.2 |
|  |  | **Low** | 55.1 ± 7.7 | 7.8 ± 5.8 | 85.9 |
|  | **160** | **Top** | 56.1 ± 2.6 | 21.2 ± 1.8 | 62.1 |
|  |  | **Mid** | 58.5 ± 2.2 | 16.0 ± 9.6 | 72.7 |
|  |  | **Low** | 55.5 ± 4.5 | 12.2 ± 16.7 | 78.1 |
|  | **240** | **Top** | 50.4 ± 4.2 | 46.1 ± 2.8 | 8.4 |
|  |  | **Mid** | 59.9 ± 2.0 | 39.9 ± 5.3 | 33.5 |
|  |  | **Low** | 60.5 ± 2.2 | 32.2 ± 9.1 | 46.8 |

## Supplementary Figures


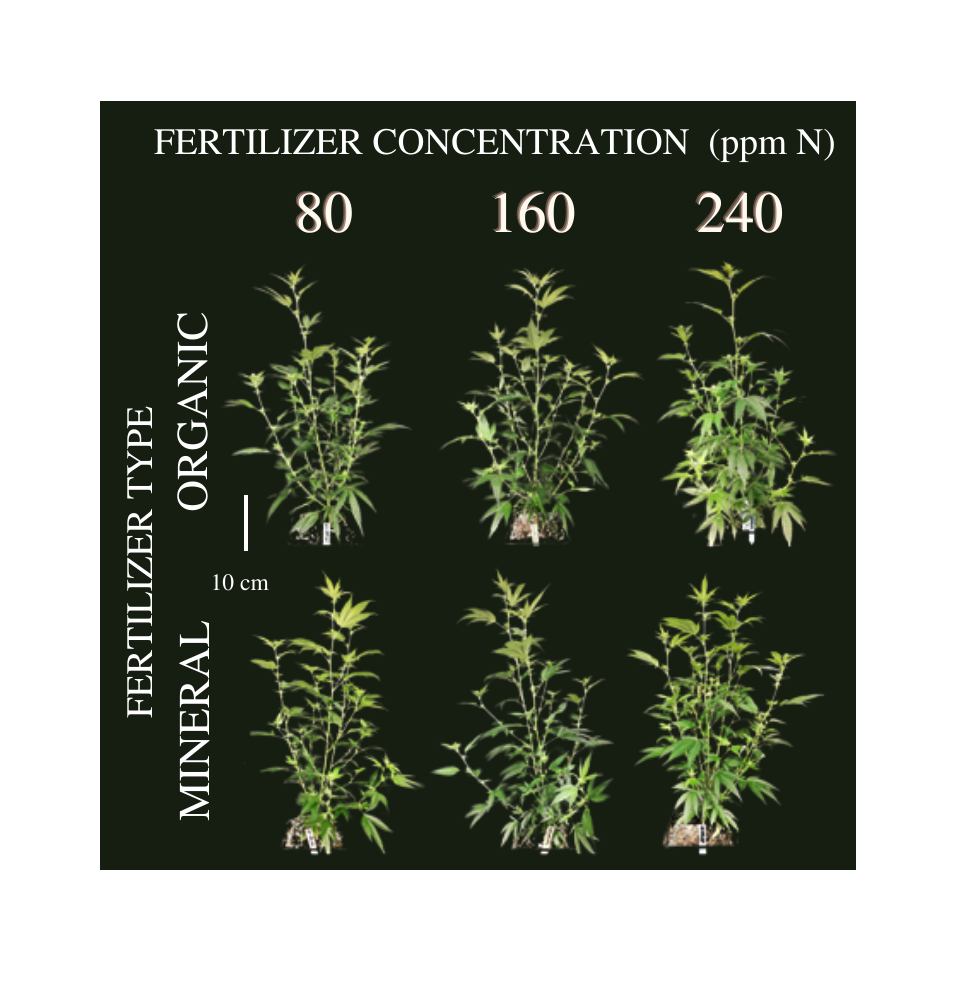


Supplementary Figure 1**.** Exemplary plants at the end of the vegetative stage (36 DAP).
